# Metformin Alleviates Liver Metabolic Dysfunction in Polycystic Ovary Syndrome by Activating the Ethe1/Keap1/PINK1 Pathway

**DOI:** 10.7150/ijbs.104778

**Published:** 2025-05-21

**Authors:** Yuan Xie, Ying Tian, Junting Huang, Wanying Deng, Xiaohui Li, Yujia Liu, Hao Liu, Lei Gao, Qiu Xie, Qi Yu

**Affiliations:** 1 Department of Obstetrics and Gynecology, National Clinical Research Center for Obstetric & Gynecologic Diseases, State Key Laboratory for Complex Severe and Rare Diseases, Peking Union Medical College Hospital, Chinese Academy of Medical Sciences & Peking Union Medical College, Peking Union Medical College Hospital (Dongdan Campus), No.1 Shuaifuyuan Wangfujing Dongcheng District, Beijing, 100730, China.; 2 Center of Reproductive Medicine, Department of Obstetrics and Gynecology, Shengjing Hospital of China Medical University, Shenyang, China.; 3 Eight-year Medical Doctor Program, Chinese Academy of Medical Sciences and Peking Union Medical College, Beijing 100730, China.; 4 Department of Child and Adolescent Health, Public Health College, Harbin Medical University, Harbin 150081, China.; 5 Key Laboratory of Epigenetic Regulation and Intervention, Institute of Biophysics, Chinese Academy of Sciences, Beijing, 100101, China; 6 Department of Medical Research Center, State Key Laboratory for Complex Severe and Rare Diseases, Peking Union Medical College Hospital, Chinese Academy of Medical Science and Peking Union Medical College, Beijing 100730, China.

**Keywords:** Metformin, Ethe1, Metabolic Dysfunction-associated Fatty Liver Disease, Mitochondrial Dysfunction, Mitophagy, Polycystic Ovary Syndrome

## Abstract

**Background:** Polycystic ovary syndrome (PCOS) is a reproductive endocrine disease characterized by metabolic abnormalities, with 34-70% of patients with PCOS also presenting non-alcoholic fatty liver disease (NAFLD). Metformin is a first-line treatment for relieving insulin resistance in PCOS; however, the potential therapeutic application of metformin for preventing NAFLD/metabolic dysfunction-associated fatty liver disease (MAFLD) in PCOS remains under-explored. Here, we investigated the potential protective effects and the underlying mechanisms of metformin against hepatic lipid metabolic disorders in prenatal anti-Müllerian hormone (PAMH)-induced PCOS mice.

**Methods:** First, we developed a prenatal AMH-induced PCOS-like model using pregnant C57BL/6N mice. Female offspring of mice were then subjected to the glucose tolerance test and insulin tolerance test pre- and post-treatment with metformin. H&E staining, serum hormone, and biochemical analyses were performed to determine the effects of metformin on metabolic abnormalities and liver damage in the PCOS-like model. To verify the specific mechanism of action of metformin, dehydroepiandrosterone (DHEA) and free fatty acids (FFAs; palmitic acid and oleic acid) induced alpha mouse liver 12 (AML-12) cells were used to establish a mouse liver cell model of adipose-like degeneration and lipid deposition.

**Results:** Metformin effectively alleviated hepatic lipid accumulation in the PCOS mice. Furthermore, mitochondrial dysfunction and loss of redox homeostasis in the liver of PCOS mice were rescued upon metformin administration. Mechanistic insights reveal that metformin regulates mitochondrial autophagy in PCOS liver tissue via the activation of the Ethe1/Keap1/Nrf2/PINK1/Parkin pathway, thereby improving liver recovery in PCOS mice.

**Conclusions:** Our findings highlight the role and mechanism of metformin in ameliorating abnormal mitophagy and lipid metabolic disorders in the PCOS mice livers and the potential of metformin for addressing NAFLD in PCOS mice.

## Introduction

Polycystic ovary syndrome (PCOS) is a common reproductive endocrine disease affecting 4-21% of women of reproductive age. PCOS is associated with an increased risk of metabolic syndrome, type 2 diabetes, cardiovascular diseases, mood disorders, endometrial cancer, and non-alcoholic fatty liver disease (NAFLD) 1. NAFLD is the leading chronic liver disease worldwide, affecting approximately one-third of the global adult population and placing remarkable health and economic burdens on society 1. The estimated prevalence of NAFLD varies significantly, from approximately 32% in the general population to 40-70% in individuals with type 2 diabetes 2. NAFLD and PCOS often co-occur 3; the prevalence of NAFLD in patients with PCOS is approximately 34-70%, which is markedly higher than that in the general population 4. Even after adjusting for body mass index, the prevalence of NAFLD among patients with PCOS is still 2.2-4.3-fold higher than that among controls 5. A new definition for hepatic steatosis, metabolic dysfunction-associated fatty liver disease (MAFLD), was proposed in 2020 6. The diagnostic criteria for this disease include imaging, serological, or histological evidence of fatty liver disease alongside one of the following three criteria: overweight/obesity, type 2 diabetes mellitus, or metabolic disorders, regardless of alcohol consumption.

NAFLD is more common in women with PCOS, specifically those who have high blood levels of testosterone, obesity, and insulin resistance. It is hypothesized that simple steatosis is insufficient to trigger NAFLD 7. Thus, multiple parallel factors, including obesity, oxidative stress, insulin resistance, inflammation, mitochondrial dysfunction, and hyperandrogenism, contribute to the heterogeneity of NAFLD, which has led to the currently accepted “multiple-hit hypothesis” 3,7-9. A growing amount of research suggests that the pathogenesis and clinical characteristics of these two metabolic illnesses are closely related. The metabolic disorders of PCOS and NAFLD are both impacted by insulin resistance and the subsequent hyperinsulinemia 10.

Oxidative stress plays a central role in most chronic liver diseases, particularly MAFLD 11-14. The mechanisms leading to hepatocellular fat accumulation through mitochondrial and endoplasmic reticulum dysfunction amplify reactive oxygen species (ROS) production, lipid peroxidation, and malondialdehyde (MDA) formation and influence the release of chronic inflammation and liver damage biomarkers, such as pro-inflammatory cytokines 15. A close relationship exists between increased oxidative stress and mitochondrial dysfunction and impaired autophagy, which is an important factor in MAFLD, especially during the early stages of liver fat accumulation, fatty infiltration, and liver inflammation 16,17. Impaired autophagy is also closely associated with reproductive hormones 18.

Nuclear factor erythroid 2-related factor 2 (NRF2; also known as NFE2-like bZIP transcription factor 2 (NFE2L2)) is a transcription factor that plays crucial roles in protecting cells from oxidative and xenobiotic stress by orchestrating the expression of cytoprotective genes. Under steady-state conditions, NRF2 is negatively regulated by Kelch-like epichlorohydrin-associated protein 1 (KEAP1), which promotes the ubiquitin-dependent degradation of NRF2. During oxidative stress, NRF2 is released from KEAP1 and translocates to the nucleus to activate a series of antioxidant and detoxification genes 19. Following nuclear translocation, Nrf2 can bind to specific sites of the PINK1 promoter, ultimately enhancing the transcription of PINK1 and balancing mitophagy by activating the Nrf2/PINK1 signaling pathway 20.

To date, no specific pharmacological treatment has been approved for NAFLD in PCOS 21; the existing available treatment is a change of lifestyle behaviors 22. The 2023 International Evidence-Based Guideline for the Assessment and Management of Polycystic Ovary Syndrome still recommends metformin as the first-line treatment for relieving insulin resistance in patients with PCOS 23. Notably, metformin is highly effective at managing type 2 diabetes mellitus 24 and has significant therapeutic benefits for various organs, particularly the liver 25. Furthermore, metformin can improve insulin resistance and dyslipidemia, which are commonly associated with NAFLD in PCOS 25,26. While current results are highly promising, indicating that its therapeutic properties could extend beyond managing diabetes 27, more studies are needed to fully determine the safety and effectiveness of metformin in liver disease.

Accordingly, this study aimed to explore the potential protective role and regulatory mechanism of metformin in the liver with MAFLD/NAFLD in PCOS mice. Our findings revealed the role and mechanism of metformin in alleviating abnormal mitophagy and lipid metabolic disorders in the PCOS liver and emphasized the potential of metformin for addressing NAFLD in PCOS.

## Methods

### Study design and animal experiments

This study was performed to elucidate the potential protective role and regulatory mechanism of metformin in the livers of patients with MAFLD/NAFLD and PCOS. Female C57BL/6N mice (six to eight weeks old) were obtained from the Beijing Vital River Laboratory Animal Technology Corporation. All experimental procedures adhered to the National Institutes of Health's Guide for the Care and Use of Laboratory Animals. Mice were allowed to acclimatize for 1 week before commencement of the experiments, housed under specific pathogen-free conditions. To develop a prenatal anti-Müllerian hormone (PAMH)-induced PCOS-like model, timed pregnant adult C57BL/6N dams (12-16 weeks old) received daily intraperitoneal (i.p.) injections from embryonic day (E) 16.5 to E18.5. Each F0 dam was administered 200 μL of a solution containing 0.12 mg/kg/day of human AMH (Müllerian-inhibiting factor/AMH, Mouse (P. pastoris, His), MCE, HY-P71712), as previously reported 28. Control F0 dams were administered phosphate-buffered saline (PBS). Female F1 offspring were weighed every 10 days post-birth to monitor their weight gain. At 2 months old, vaginal smears were taken daily for 7 days to assess sexual maturity. Glucose metabolism was evaluated using an oral glucose tolerance test (ipGTT) and insulin tolerance test (ipITT). A subset of these mice was randomly selected to receive metformin, which was administered intragastrically (i.g.) at high (250 mg/kg/day), medium (50 mg/kg/day), and low (10 mg/kg/day) doses. Treatment was performed for 2 months. Post-treatment, vaginal smears and ipGTT and ipITT were repeated to determine the most effective dose. At 4 months old, female F1 offspring were placed in metabolic chambers to evaluate respiratory and motor metabolism. Throughout the study, mice were housed in a temperature-controlled environment with free access to food and water under a standard light-dark cycle, housed with up to six mice per cage. All procedures received approval from the Committee on the Ethics of Animal Experiments of the Peking Union Medical College Hospital (XHDW-2023-138).

### Metabolic measurements

Mice were individually housed in metabolic chambers of the Comprehensive Lab Animal Monitoring System (Columbus Instruments, Columbus, OH, USA) with free access to water and normal chow diet (NCD). After a 24-h acclimation period, energy expenditure was measured over the next 24 h. Heat production, locomotor activity, and rates of oxygen consumption (VO_2_) and carbon dioxide production (VCO_2_) were assessed using the Oxymax system (Columbus Instruments). VO_2_, VCO_2_, and heat production were measured every 5 min and normalized to body weight. The respiratory exchange ratio (RER) was calculated as the ratio of VCO_2_ to VO_2_. Locomotor activity was quantified by counting interruptions in the infrared beams, specifically the total number of X- and Z-beam breaks.

### Oil O red staining

Fixed liver tissue was dehydrated in 15% and 30% sucrose solutions and embedded in an optimal cutting temperature compound (4583, Sakura, Tokyo, Japan). Samples were sectioned at a thickness of 8 µm. Hepatocyte steatosis *in vivo* and *in vitro* was visualized using the Modified Oil Red O Stain Kit (G1261, Solarbio, Beijing, China) and Oil Red O Stain Kit for Cultured Cells (G1262, Solarbio, Beijing, China), respectively.

### Measurement of malondialdehyde (MDA), superoxide dismutase (SOD), and glutathione (GSH) / glutathione oxidized (GSSG) content

MDA content is an important parameter for measuring the potential antioxidant capacity of the body, which can indicate the rate and intensity of lipid peroxidation and indirectly reflects the degree of tissue peroxidation damage 29. Superoxide dismutase (SOD) is an antioxidant enzyme in living organisms that catalyzes the dismutation of superoxide anion radicals to produce oxygen and hydrogen peroxide, which indirectly reflects the ability of the body to scavenge free radicals 30. Protein concentrations in liver tissue and AML-12 cells were determined using a BCA Protein Assay Kit (P0012, Beyotime). Colorimetric assays were conducted (MDA, BC0025, Solarbio; SOD, BC0175, Solarbio; and GSH/GSSG, S0053, Beyotime) according to the respective manufacturers' instructions.

### Evaluation of ATP content

ATP concentration was analyzed using firefly luciferase, where fluorescence production is proportional to ATP concentration. The liver tissue was covered with lysis buffer from the ATP assay kit (BC0305, Solarbio) and centrifuged at 12,000 ×*g* for 5 min (4 °C). The supernatant was collected and used for subsequent assays. All samples were kept on ice to reduce enzymatic ATP hydrolysis. ATP concentration was measured using a luminometer (BioTek, Synergy H1).

### Measurement of mitochondrial complex activity

The Mitochondrial Complex I Activity Assay Kit (BC0515, Solarbio), Mitochondrial Complex II Activity (BC3235, Solarbio), Mitochondrial Complex III Activity Colorimetric Assay (BC3245, Solarbio), Mitochondrial Complex IV Activity Assay Kit (BC0945, Solarbio), and Mitochondrial Respiratory Chain Complex V Activity (BC1445, Solarbio) were used to evaluate the mitochondrial complex activity of liver tissues.

### Cell culture and transfection

Alpha mouse liver 12 (AML-12) cells were purchased from the National Biomedical Experimental Cell Resource Institute. Cells were cultured in DMEM containing 4.5 g/L D-glucose, 110 mg/L sodium pyruvate, L-glutamine (C11995500BT, Gibco), and 10% FBS (10099141, Gibco), at 37 ºC in a 5% CO_2_ incubator. Cells were seeded at a density of 2 × 10^5^ viable cells·mL^-1^ and passaged every 3 days.

For transient transfections, cells were grown to 70-90% confluence and transfected using Lipofectamine-3000 (L3000015, ThermoFisher Scientific) transfection reagents according to the manufacturer's instructions. Briefly, the lipofectamine mix and siRNA mix were prepared separately, mixed, and incubated for 10 min at 20-25 ºC before addition to the cells in Opti-MEM® medium. The transfected cells were incubated at 37 °C and 5% CO_2_ for 48 h before harvesting.

### Ethe1, Nrf2, and PINK1 knockdown using small interfering RNA (siRNA)

AML-12 cells (0.3 × 10^5^ /ml) were plated onto six-well plates and transfected with siRNAs after 24 h. The sequence information of the siRNAs is presented in Table 1.

### RNA extraction and real-time quantitative PCR (RT-qPCR) analysis

The mRNA expression levels of mtDNA, Ethe1, PINK1, and Nrf2 were determined using RT-qPCR. At 48 h after siRNA transfection, total RNA was extracted from cells using TRIzol (15596018, Gibco) according to the manufacturer's instructions. Reverse transcription was performed using TransScript First-Strand cDNA Synthesis SuperMix (AT301-02, TransGen Biotech, China). RT-qPCR was performed using the Fast SYBR Mixture (Low ROX) (CW2621M, CWBIO, China). The PCR primer sequences are shown in Table 2.

### Colocalization of LC3/PINK1 and Mito-Tracker Red

MitoTracker Red-labeled AML-12 cells were fixed in 4% paraformaldehyde for 10 min at 20-25 °C, treated with 0.1% Triton-X100 for 20 min and 5% BSA for 1 h, and incubated with anti-LC3B (1:500, ab192890, Abcam) and anti-PINk1 (1:500, 23274-1-AP, Proteintech) overnight at 4 °C. Thereafter, the cells were washed using PBS and incubated with secondary antibodies. The nuclei were counter-stained with DAPI (C1002, Beyotime, China). The co-localization of LC3 and Mito-Tracker Red was observed using confocal microscopy.

### Co-immunoprecipitation and western blot analysis

Co-immunoprecipitation (Co-IP) was performed using the extracted liver tissue proteins. The extracted proteins were purified using Pierce protein A/G agarose 28. Following loading of the Pierce protein A/G agarose resin onto the spin column, 10 µg of ETHE1 persulfide dioxygenase antibody (B-12) (ETHE1, sc-393869, Santa Cruz Biotechnology) was added to the column and washed. The cross-linked Flag antibody-bound spin column was retained. Pre-treated protein lysates with control agarose resin were added to the column and incubated overnight at 4 ºC. Finally, the columns were washed three times, and the flow-through was collected for western blot analysis.

The input and IP proteins were dissolved in sodium dodecyl sulfate-polyacrylamide gel electrophoresis (SDS-PAGE) sample buffer. Equal amounts of the protein (10 µg) were separated via 10% SDS-polyacrylamide gel electrophoresis and transferred onto PVDF membranes. The membranes were then washed with Tris-buffered saline, blocked for 1 h at 20-25 ºC, and incubated with the following appropriate primary antibodies: anti-βactin (1:5000; AC026, Abclonal), anti-Ethe1 (1:2000, 27786-1-AP, Proteintech), anti-ETFDH polyclonal (1:2000, 11109-1-AP, Proteintech), and anti-Keap1 (1:5000, 10503-2-AP, Proteintech). After washing, the cells were incubated with a secondary antibody (horseradish peroxidase-conjugated goat anti-rabbit (1:5000, ZB2301, Zsbio) and horseradish peroxidase-conjugated goat anti-mouse (1:5000, ZB2305, Zsbio) for 1 h at 20-25 °C. Finally, the blots were visualized using SuperSignal-enhanced chemiluminescent substrate solution (WBULS0100, Millipore).

### Western blot analysis

Liver tissue or cells were lysed using RIPA buffer (R0010, Solarbio) with PMSF (1:100, P0100, Solarbio). Samples were centrifuged to obtain the protein supernatant. All protein samples were dissolved in 5× SDS-PAGE loading buffer (P1040, Solarbio), heated in a 100 ℃ water bath for 10 min, and stored. The membranes were then blocked with 5% skim milk powder dissolved in TBST for 1 h at room temperature. The PVDF membranes were incubated with anti-Ethe1 (1:2000, 27786-1-AP, Proteintech), anti-ETFDH polyclonal (1:2000, 11109-1-AP, Proteintech), NRF2,NFE2L2 polyclonal antibody (1:5000, 16396-1-AP, Proteintech), anti-Keap1 (1:5000, 10503-2-AP, Proteintech), PARK2/Parkin polyclonal antibody (1:2000, 14060-1-AP, Proteintech), PINK1 polyclonal antibody (1:500, 23274-1-AP, Proteintech), MFN2 polyclonal antibody (1:5000, 12186-1-AP, Proteintech), DRP1 (C-terminal) polyclonal antibody (1:2000, 12957-1-AP, Proteintech), anti-TOMM20 antibody (1:2000, ab186735, Abcam), VDAC1/Porin polyclonal antibody (1:2000, 55259-1-AP, Proteintech), P62, SQSTM1 polyclonal antibody (1:10000, 18420-1-AP, Proteintech), anti-LC3B antibody (1:2000, ab192890, Abcam), anti-βactin (1:5000; AC026, Abclonal), COXIV polyclonal antibody (1:5000, 11242-1-AP, Proteintech), and Histone H3 polyclonal antibody (1:5000, 17168-1-AP, Proteintech) at 25 °C for 2 h. After incubation with the corresponding secondary antibody (horseradish peroxidase-conjugated goat anti-rabbit (1:5000, ZB2301, Zsbio) and horseradish peroxidase-conjugated goat anti-mouse (1:5000, ZB2305, Zsbio)) for 1 h at 20-25 °C, the blots were visualized using SuperSignal-enhanced chemiluminescent substrate solution (WBULS0100, Millipore).

### Statistical analysis

The data are presented as mean ± SD and were analyzed using GraphPad Prism software (version 8.0.1.244; GraphPad Software Inc., San Diego, CA, USA). Statistical significance was evaluated using one-way analysis of variance (ANOVA) with IBM SPSS Statistics software (version 22.0). Differences between groups were assessed using a two-tailed unpaired Student's t-test, one-way ANOVA, or two-way ANOVA depending on the specific conditions. A *p*-value < 0.05 was considered statistically significant. Each experiment was replicated at least three times.

Data management was conducted using Microsoft Excel 2019. Normality and homogeneity of variances were assessed before statistical analyses. For parametric tests, such as unpaired t-tests (two-tailed) for two-group comparisons and one-way ANOVA for multiple-group comparisons, normality and homogeneity of variances were confirmed. For non-normally distributed data or heterogeneity of variances, appropriate non-parametric tests or transformations were applied. Multiple comparisons were adjusted using suitable methods, such as Bonferroni or Benjamini-Hochberg correction, to mitigate the risk of Type I errors. Correlation analyses were also conducted to explore the relationships between variables, performed using GraphPad Prism 9 software. The significance levels are denoted as follows: *p < 0.05; **p < 0.01; ***p < 0.001; and ****p < 0.0001.

A correlation heat-map was created by calculating the correlation coefficients for all variable pairs, ranging from -1 to 1, where values close to 1 indicate a strong positive correlation, values close to -1 indicate a strong negative correlation, and values close to 0 indicate little to no correlation. The correlation matrix was then visualized as a heat-map, with colors ranging from cool tones (blue) for negative correlations to warm tones (red) for positive correlations.

## Results

### Analysis of the PCOS phenotype in a PAMH-induced PCOS mouse model

**Figure 1:**
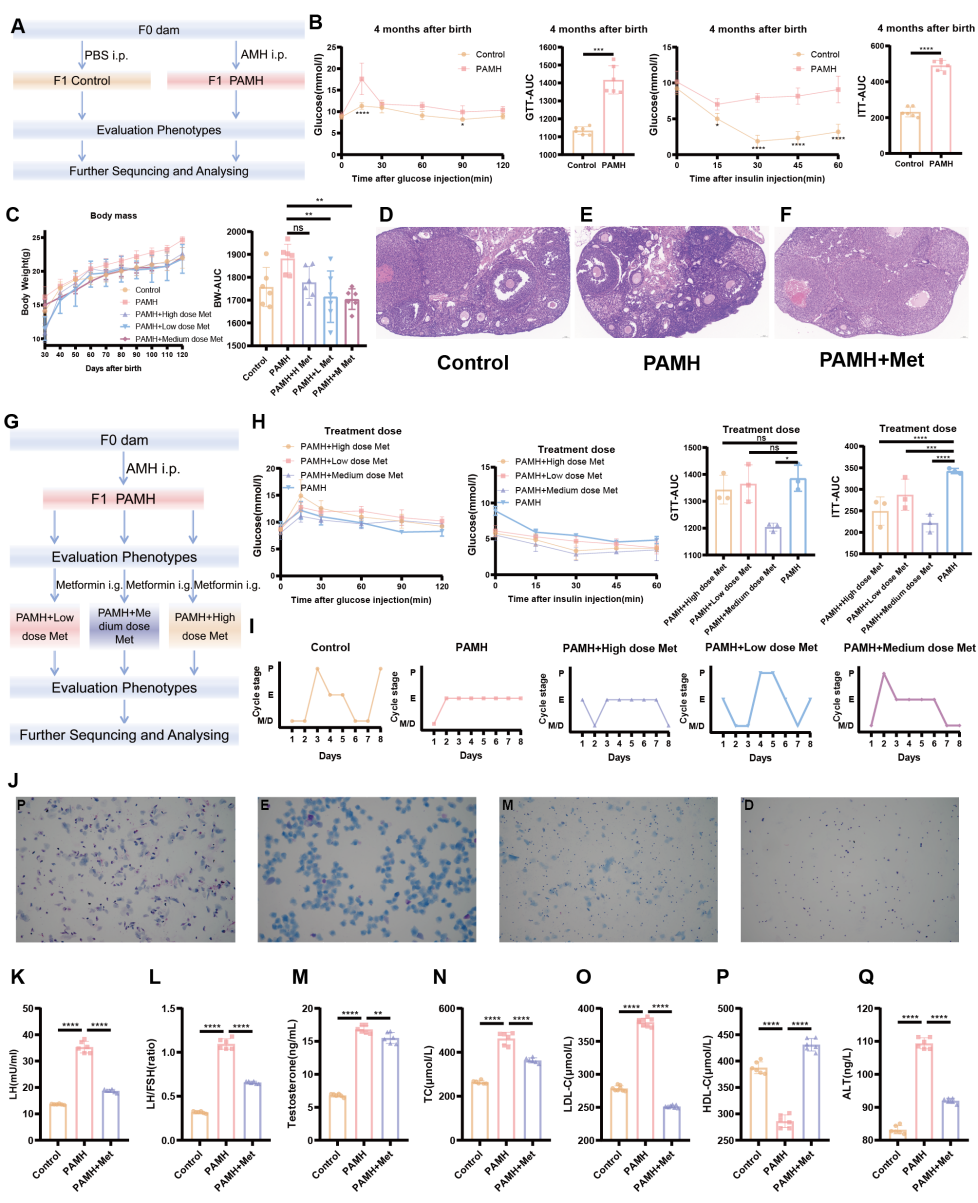
** Phenotypic evaluation of normal and PAMH-induced mice showing that metformin treatment alleviated metabolic and reproductive abnormalities. (A)** PAMH-induced PCOS-like mouse model and metformin treatment. **(B)** Glucose tolerance and insulin resistance in 4-month-old female mice for control and PAMH groups. **(C)** Comparison of body weight curves and AUC of body weight changes in control mice and before and after metformin oral administration in PAMH-induced PCOS-like mice. **(D-F)** Ovarian morphology by H&E staining in control, PAMH-induced, and metformin administered mice. **(G)** Flowchart of setting-up the dose-exploration experiment in PAMH mice. **(H)** Comparison of ipGTTs and ipITTs between the PAMH and metformin treated groups. **(I)** Representative estrous cyclicity assessment 4 four-month-old female mice for 8 consecutive days by vaginal cytology. **(J)** Representative cycles for five groups where each dots represent one day; M, metestrus; D, diestrus; P, proestrus; E, estrus. **(K-Q)** Serum concentrations of luteinizing hormone (LH) **(K)**, LH/FSH** (L)**, testosterone **(M)**, total cholesterol (TC) **(N)**, low-density lipoprotein cholesterol (LDL-C)** (O)**, high-density lipoprotein cholesterol (HDL-C)** (P)**, and alanine transaminase (ALT)** (Q)** as measured by ELISA (n = 6/group). All error bars are mean values ± SD, *p*-values were determined by unpaired two-tailed Student's t test (*n* = 3) in independent biological experiments. *p < 0.05; **p < 0.01; ***p < 0.001; ****p < 0.0001.

To determine the role of NAFLD in PCOS, a mouse model of PAMH-induced PCOS was established according to previous studies 28,31. Body weight, estrous cycle, sex hormone levels, liver function, lipid levels, and ovarian/liver tissue morphology were monitored. Figure 1A outlines the procedure used to establish the PAMH-induced PCOS mouse model and the treatment schedule with metformin.

As the abnormal metabolism of PCOS and NAFLD is both influenced by insulin resistance, we examined whether there were any defects in glucose metabolism for PAMH-induced PCOS mice. Four-month-old PCOS mice and control mice were subjected to GTT and ITT assays (Figure 1B). Blood glucose levels were found to significantly differ between the two groups at 15 min after glucose or insulin injection (*p* < 0.0001 and *p* = 0.0119, respectively). The area under the curve (AUC) values of the GTT and ITT curves in the PCOS model group were significantly higher than those in the control group (*p* = 0.0005 and *p* < 0.0001, respectively) (Figure 1B). These results suggest that PAMH-induced PCOS mice exhibit glucose metabolism defects, including insulin resistance. To further explore the effect of metformin on PAMH mice, three concentrations (high: 250 mg/kg/day; medium: 50 mg/kg/day; low: 10 mg/kg/day) were established based on the time and concentration reported in the literature for preliminary exploration 32-34. The flowchart of setting-up the dose-exploration experiment in the PAMH mice is shown in Figure 1G. Treatment with metformin was initiated two months after birth and continued for 2 months. Finally, the efficacy of metformin was evaluated based on body weight, estrous cycle, GTTs, and ITTs. The weight of PAMH mice was higher than that of control mice based on the AUC (*p* = 0.0154). The body weight of PAMH mice significantly decreased after treatment with low and medium metformin doses compared with that of untreated PAMH mice (*p* = 0.0049 and *p* = 0.0027, respectively) (Figure 1C). Furthermore, the AUC values of the GTT and ITT for PAMH mice treated with a medium dose of metformin were lower than those for PAMH mice (*p* = 0.0102 and *p* = 0.0025, respectively) (Figure 1H).

We also examined the reproductive system and liver phenotypes in the PAMH mice. Figure 1I shows normal estrous cycles in the control mice, whereas abnormal cycles were displayed in the PAMH mice. After two months of medium-dose metformin treatment, the estrous cycles of PAMH mice were improved and similar to those of normal mice. Representative images of the estrous cycle are shown in Figure 1J. H&E staining (Figure 1D-F), serum hormone, and biochemical (Figure 1K-Q and Figure S2A-D) results also reveal that the PAMH-induced model mimicked the reproductive abnormalities and impaired liver function phenotypes of PCOS, and metformin treatment alleviated these abnormalities. More detailed results of the GTT and ITT evaluation in the PCOS model mice are shown in Figure S1A-T and described in the Supplementary Information.

Collectively, PAMH-induced mice exhibit PCOS-like characteristics with insulin resistance and functional liver defects, and metformin treatment can effectively improve these phenotypes.

### Metformin alleviates metabolic abnormalities in PAMH-induced PCOS-like mice

**Figure 2:**
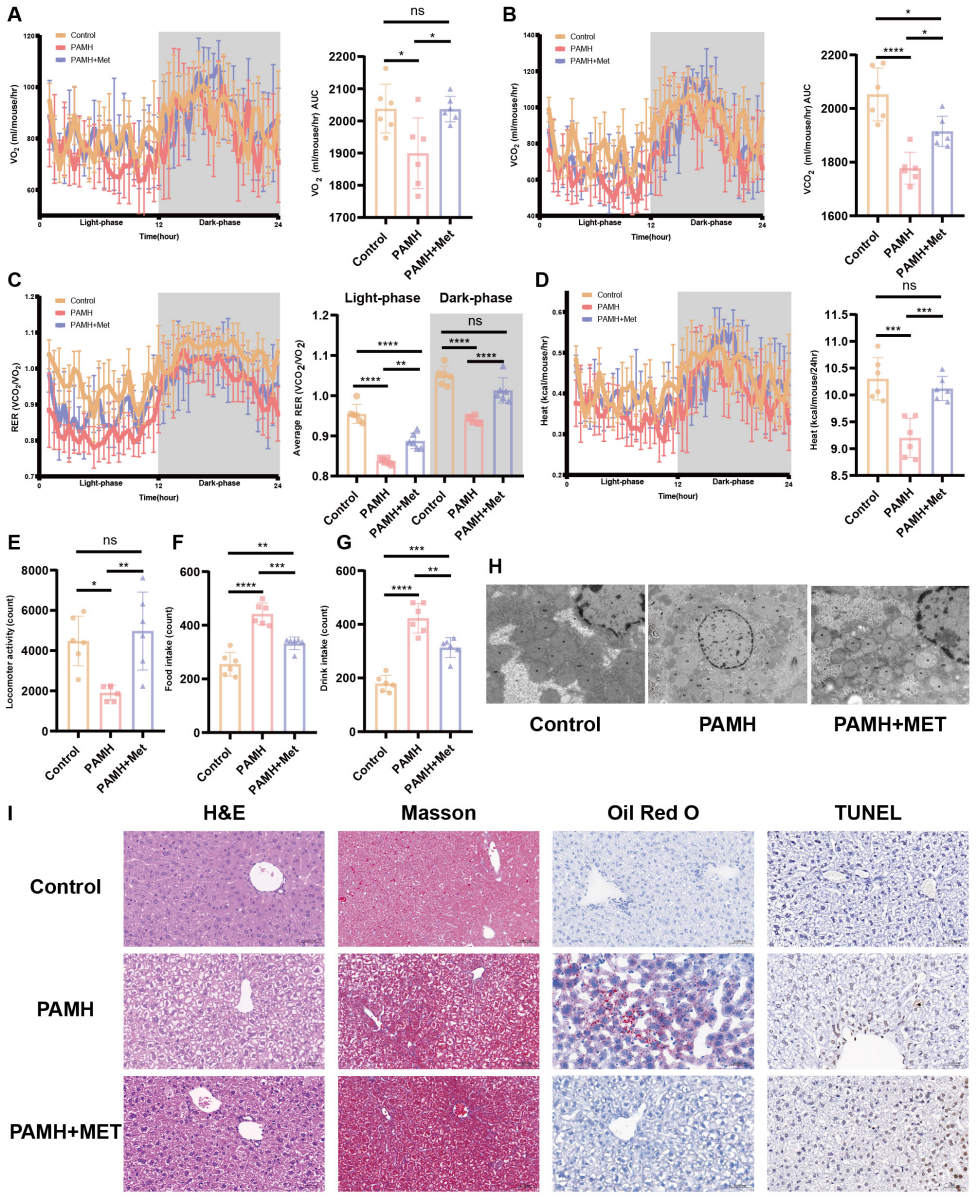
** Metformin alleviated metabolic abnormalities and liver damage in a PAMH-induced PCOS-like mouse model. (A-G)** Metabolic state of mice based on respiration **(A-C)**, basal metabolism **(D)**, movement **(E)**, and food and drink intake **(F,G)** (*n* = 6/group). **(H)** Transmission electron microscopy images of mice liver tissues in the control, PAMH-induced, and metformin-administered groups. **(I)** Liver morphology by H&E staining, Masson's staining, Oil Red O staining, and TUNEL assay. All error bars are mean values ± SD, *p-*values were determined by unpaired two-tailed Student's t test (*n* = 3) in independent biological experiments. *p < 0.05; **p < 0.01; ***p < 0.001; ****p < 0.0001.

PCOS is often accompanied by NAFLD 3, which may be related to the widespread metabolic abnormalities of PCOS. We performed metabolic testing in PAMH mice. Figure 2A-G shows the metabolic state of the mice. Each mouse was placed in a metabolic chamber to indirectly measure respiration, movement, and basal metabolism. Indirect calorimetry indicated that metformin administration rescued the oxygen consumption, carbon dioxide production, the respiratory exchange ratio, and heat production in PAMH-induced PCOS-like mice during both light and dark cycles (Figure 2A-D). The PAMH mice consumed more water and food and had lower locomotor activity than the control mice. Metformin improved the eating and exercise habits of the PAMH mice to some extent (Figure 2E-G). Taken together, metformin treatment alleviated widespread metabolic abnormalities in PAMH-induced PCOS-like mice.

### Metformin improves mitochondrial function in the liver of PAMH-induced PCOS mice

Homeostasis of mitochondrial function is vital to various metabolic processes, and increased oxidative stress level indicates mitochondrial abnormalities. Accordingly, we further evaluated mitochondrial function in each group of mice. Figure 2 (H and I) highlights the damage to the mitochondrial structure, autophagosome reduction, hepatocyte steatosis, and lipid deposition in the liver tissues of the PCOS mice, which could be mitigated by treatment with metformin.

**Figure 3:**
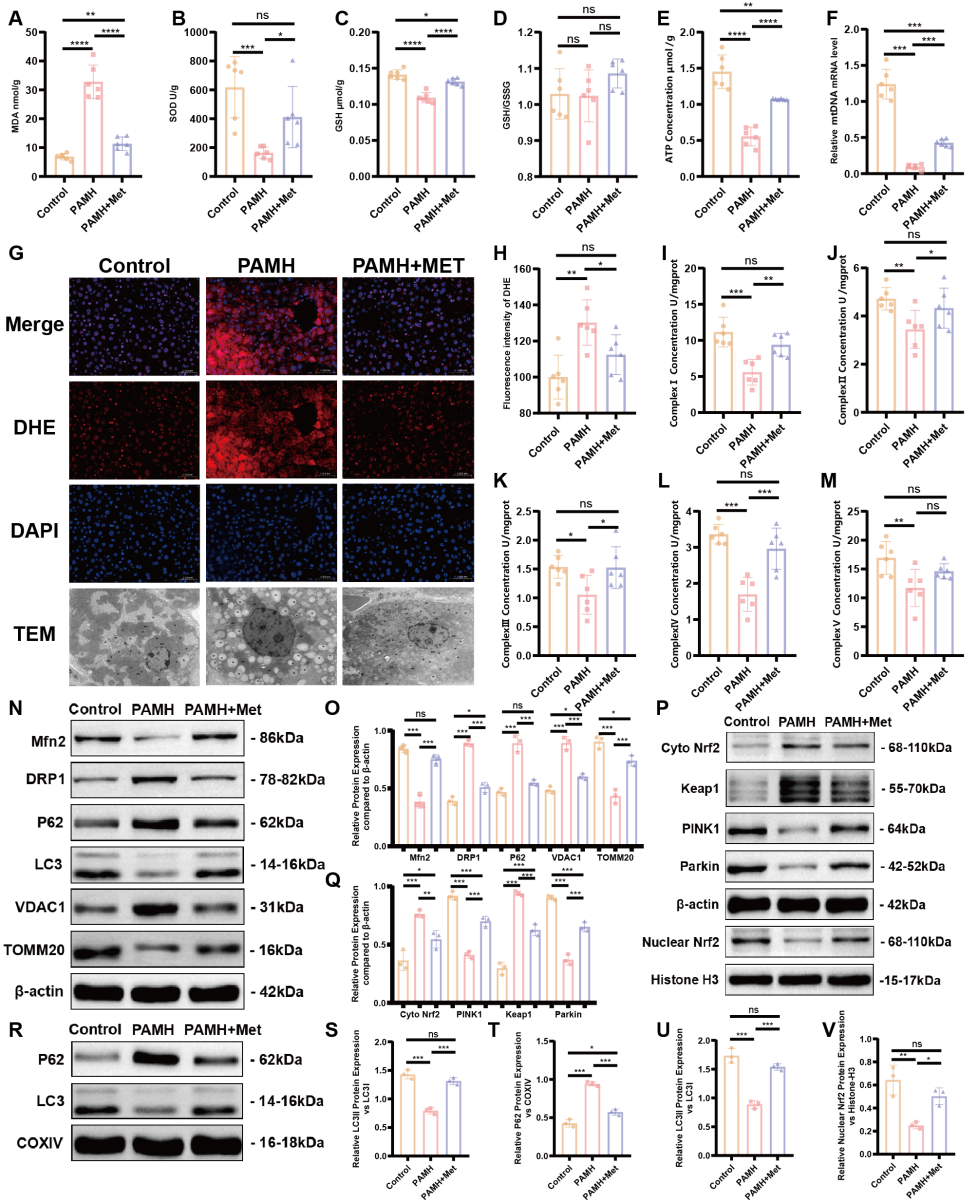
** Metformin improves mitochondrial function in the liver of PAMH-induced PCOS mice.** Malondialdehyde (MDA) **(A)**, superoxide dismutase (SOD) **(B)**, glutathione (GSH) **(C)**, GSH/GSSG **(D)**, ATP** (E)**, and mitochondrial DNA (mtDNA) **(F)** in liver tissues from the control, PAMH-induced, and metformin treatment groups. **(G, H)** Reactive oxygen species levels in liver tissues labeled with dihydroethidium (DHE) as observed under a fluorescence microscope **(G)**. Mean fluorescence intensity of DHE in each group **(H)**. **(I-M)** Mitochondrial respiratory chain complexes: Complex I (NADH dehydrogenase) **(I)**, Complex II (succinate-coenzyme Q reductase) **(J)**, Complex III (ubiquinol-cytochrome c oxidoreductase) **(K)**, Complex IV (cytochrome C oxidase) **(L)**, and Complex V (ATP synthase) **(M)**. **(N-Q, S, V)** Markers related to mitochondrial autophagy (Mfn2, DRP1, LC3, P62, TOMM20, VDAC1, Nrf2, Keap1, PINK1, and Parkin) in total protein extraction from liver tissue based on western blotting **(N, P)**. Protein relative expression levels were calculated in the control group (orange bars), PAMH group (pink bars), and PAMH+Met group (purple bars) and also shown **(O, Q, S, V)**. **(R, T, U)** Expression of p62 and LC3 in each group. All error bars are mean values ± SD, *p-*values were determined by unpaired two-tailed Student's t test (*n* = 3) in independent biological experiments. *p < 0.05; **p < 0.01; ***p < 0.001; ****p < 0.0001.

As shown in Figure 3A-F, excessive accumulation of MDA was observed, and the SOD and GSH levels, ATP content, and mtDNA copy number were decreased in the liver tissues of PAMH mice. The DHE level indicated a high level of ROS in the liver tissues of PAMH mice relative to that of the PAMH+Metformin mice (Figure 3G and H). The mitochondrial respiratory chain complexes also exhibited varying degrees of mitochondrial functional reduction in the PCOS group (Figure 3I-M). These results indicate that metformin partially rescued lipid peroxidation, antioxidant and mitochondrial function, and oxidative stress level in the PCOS mice.

The occurrence of mitophagy is a key process in the maintenance of mitochondrial function. We detected markers related to mitochondrial autophagy in liver tissues (Figure 3N, O, S). The expression of mitofusin 2 (Mfn2), was found to decrease in the model group, whereas that of the mitochondrial fission protein dynamin-related protein 1 (DRP1) and voltage-dependent anion channel 1 (VDAC1) increased. Such findings indicate a disruption in the dynamic balance of the mitochondria required for mitochondrial energy metabolism.

Furthermore, the expression levels of proteins related to mitochondrial autophagy, such as microtubule-associated protein 1 light chain 3 (LC3) and translocase of the outer mitochondrial membrane 20 (TOMM20), were decreased, indicating reductions in mitochondrial autophagy and mitochondrial-mediated apoptosis, which exacerbate the disruption of mitochondrial homeostasis. Sequestosome 1/p62 is not only an adaptor protein between autophagosomes and substrates but also a selective autophagy substrate 35. We observed the up-regulation of p62 in the PCOS group. Concurrently, mitochondrial proteins were extracted from fresh liver tissues, and the expression levels of p62, LC3, Mfn2, DRP1, VDAC1, TOMM20, PINK1, and Parkin were assessed. The results aligned with those of the total liver tissue protein data (Figure 3R, T, U; Figure S6A, B). These results indirectly reflected a reduction in mitochondrial autophagy in the liver tissues of the model group, which was partially rescued by the application of Metformin.

To explore the specific mechanisms and signaling pathways that regulate mitochondrial autophagy, we focused on the expression of Keap1/Nrf2 and PINK1/Parkin according to previous studies. The expression of Keap1 was found to increase while that of Nrf2 increased in the cytoplasm and decreased in the nucleus in liver tissues of PAMH mice. Moreover, the expression levels of PINK1 and Parkin decreased in the liver tissues of PAMH mice, indicating weakened mitochondrial autophagy; however, treatment with metformin partially enhanced mitochondrial autophagy (Figure 3P, Q, V).

To summarize, mitochondrial dysfunctions, especially decreased mitophagy, were associated with metabolic abnormalities in PCOS liver tissue. Metformin treatment improved mitochondrial autophagy in PCOS liver tissue by activating the Keap1/Nrf2/PINK1/Parkin pathway.

### Bioinformatics analysis of differentially expressed genes in PCOS liver tissues

**Figure 4:**
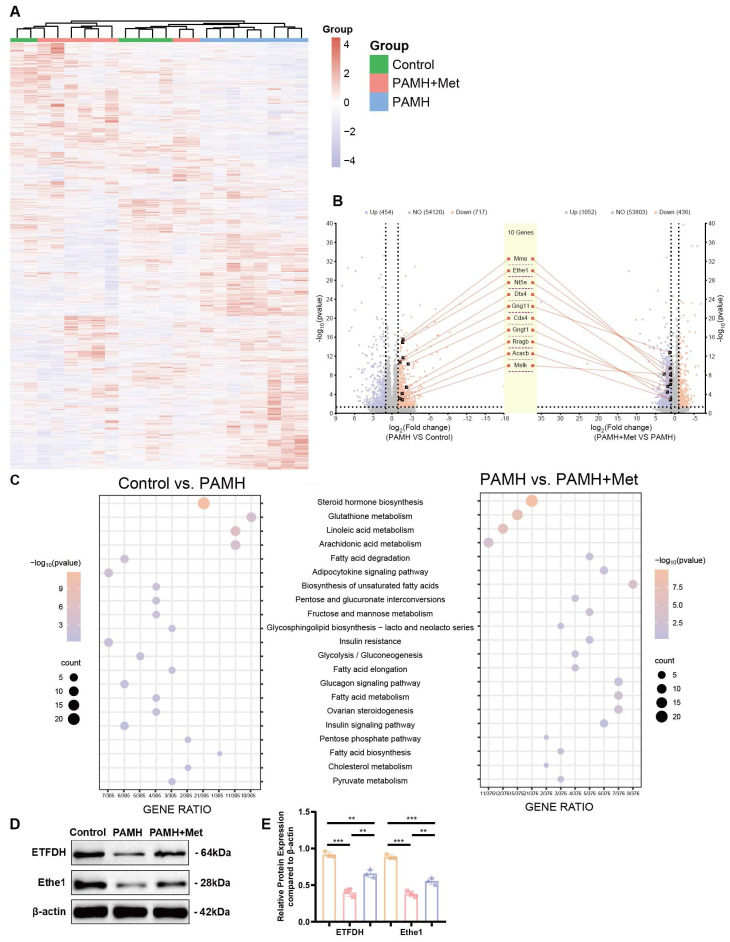
** Bioinformatics analysis of differentially expressed genes in PCOS liver tissues. (A)** Heat-map of gene expression in liver tissues of mice in each group. **(B)** Up- and downregulated genes in the PAMH group compared to the control group (left panel) and those in the PAMH+Met group compared to the PAMH group (right panel). **(C)** Common KEGG pathways of significantly differentially expressed genes both in control vs. PAMH and PAMH vs. PAMH+Met groups.** (D, E)** Protein expression levels of ETFDH and Ethe1 in liver tissues in each group. The relative expression gray value was assessed using Image J for the control group (orange bars), PAMH group (pink bars), and the PAMH+Met group (purpose bars). All error bars are mean values ± SD, *p-*values were determined by unpaired two-tailed Student's t test (*n* = 3) in independent biological experiments. *p < 0.05; **p < 0.01; ***p < 0.001; ****p < 0.0001.

As the underlying mechanism through which metformin affects Keap1 and promotes mitophagy remains unknown, we performed whole-transcriptome sequencing of liver tissues from PCOS mice, control mice, and PCOS mice treated with a medium dose of metformin. This analysis aimed to identify specific targets and mechanisms by which metformin acts in the liver of PAMH-induced NAFLD mice. Figure 4A shows a heat-map of gene expression in the liver tissues of mice in each group (*n* = 8). The results indicate that PCOS mice treated with metformin (PAMH+Met group) exhibited gene-expression patterns similar to those of control mice compared to untreated PCOS mice (the PAMH group). This suggests that metformin treatment could rescue the abnormal gene expression in PCOS mice. Figure 4B describes the up- and downregulated genes in the PAMH group compared to the control group (left panel), and those in the PAMH+Met group than in the PAMH group (right panel). There were 454 genes upregulated in the PAMH group than in the control group, and 436 genes were downregulated in the PAMH+Met group than in the PAMH group. Furthermore, 717 genes were downregulated in the PAMH group than in the control group (orange dots in the left panel of Figure 4B), and 1,052 genes were upregulated in the PAMH+Met group than in the PAMH group (purple dots in right panel of Figure 4B). KEGG pathway enrichment analysis indicated that most of the differentially expressed genes were involved in pathways related to steroid hormone biosynthesis, GSH metabolism, and sugar, lipid, and insulin metabolism (Figure 4C). To obtain a responsive RNA gene set that was down-regulated in the PAMH group and which could be restored by treatment with metformin, we intersected RNAs that were significantly down-regulated in the PAMH group relative to the control group and those that were significantly upregulated upon metformin treatment in the PAMH group. Finally, we identified 244 genes that were significantly downregulated in the PAMH group than in control group, and the expression levels of these genes were significantly restored upon metformin treatment in the PAMH group (*p <0.05* after correction).

To determine the direct relationship between metformin and the 244 identified genes, we first conducted a literature review to identify reported target genes of metformin. We retrieved 10 common target genes, namely protein kinase AMP-activated catalytic subunits alpha 1 (PRKAA1), alpha 2 (PRKAA2), beta 1 (PRKAB1), beta 2 (PRKAB2), gamma 1 (PRKAG1), gamma 2 (PRKAG2), and gamma 3 (PRKAG3); electron transfer flavoprotein dehydrogenase (ETFDH); glycerol-3-phosphate dehydrogenase 1 (GPD1); and presenilin enhancer, gamma-secretase subunit (PSENEN). Next, we performed protein-protein interaction (PPI) network analysis to investigate the interactions between these 10 common metformin target genes and the 244 identified genes. Among the proteins encoded by these genes, we identified 10 candidate proteins that interact with the proteins encoded by the 10 common target genes of metformin (middle panel in Figure 4B). These 10 candidate proteins are acetyl-CoA carboxylase beta (Acacb), caudal type homeobox 4 (Cdx4), deltex E3 ubiquitin ligase 4 (Dtx4), ethylmalonic encephalopathy protein 1 (Ethe1), G protein subunit gamma 11 (Gng11), G protein subunit gamma transducin 1 (Gngt1), membrane metalloendopeptidase (Mme), ecto-5'-nucleotidase (Nt5e), Ras-related GTP binding B (Rragb), and maternal embryonic leucine zipper kinase (Melk).

Based on our previous results and a literature review, we focused on Ethe1, which interacts with the metformin target, ETFDH. Ethe1 was downregulated > 3.2-fold (log_2_FC = -1.72, *p* < 0.00001 after correction) in the PAMH group than in the control group, and upregulated > 2.2-fold (log_2_FC = 1.15, *p* < 0.00001 after correction) in the PAMH+Met group than in the PAMH group (Figure 4B). By evaluating the protein levels of Ethe1 and ETFDH in the mouse liver tissue samples, we found that both were down-regulated in the PAMH group (Figure 4D, E), aligning with the trend observed based on whole transcriptomics. Notably, this downregulation could be rescued by metformin treatment (Figure 4D, E).

Based on these experimental results, we speculate that metformin regulates Ethe1 expression through ETFDH, affecting the expression and function of downstream mitochondrial autophagy-related molecules, thereby improving liver recovery in PAMH mice. Thus, further experiments were performed to test our hypotheses.

### Metformin regulates DHEA and FFAs-induced hepatocyte injury, reducing lipid deposition and elevating mitochondrial autophagic levels in damaged hepatocytes

**Figure 5:**
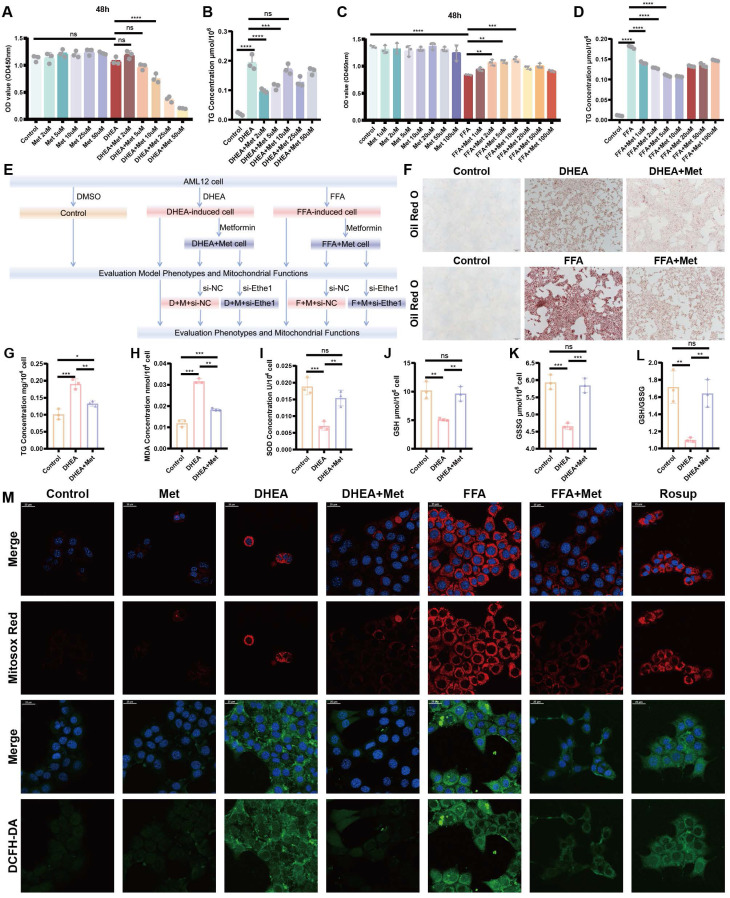
** Metformin regulates DHEA- and FFA-induced hepatocyte injury, reducing lipid deposition and oxidative stress in hepatocytes. (A-D)** Defining the optimal concentration of intervention for Met to mitigate cellular steatosis induced by DHEA** (A, B)** and FFAs** (C, D)** based on CCK8 and TG concentrations. **(E)** Flowchart of the cell experiment. **(F-L)** Representative Oil Red O staining pictures of each group. TG **(G)**, MDA **(H)**, SOD **(I)**, GSH **(J)**, GSSG **(K)**, and GSH/GSSG **(L)** concentrations in each DHEA-induced and Met-disposed group. **(M)** Each group of cells was labeled with Mitosox^TM^ Red and DCFH-DA probes and observed under a confocal microscope. All error bars are mean values ± SD, *p-*values were determined by unpaired two-tailed Student's t test (*n* = 3) in independent biological experiments. *p < 0.05; **p < 0.01; ***p < 0.001; ****p < 0.0001.

To verify the specific mechanism of action of metformin in the regulation of mitophagy by Ethe1, we induced adipose-like degeneration and established a lipid deposition-like mouse liver cell model using DHEA and FFAs. The specific design and flowchart of these cell experiments are shown in Figure 5E.

**Figure 6:**
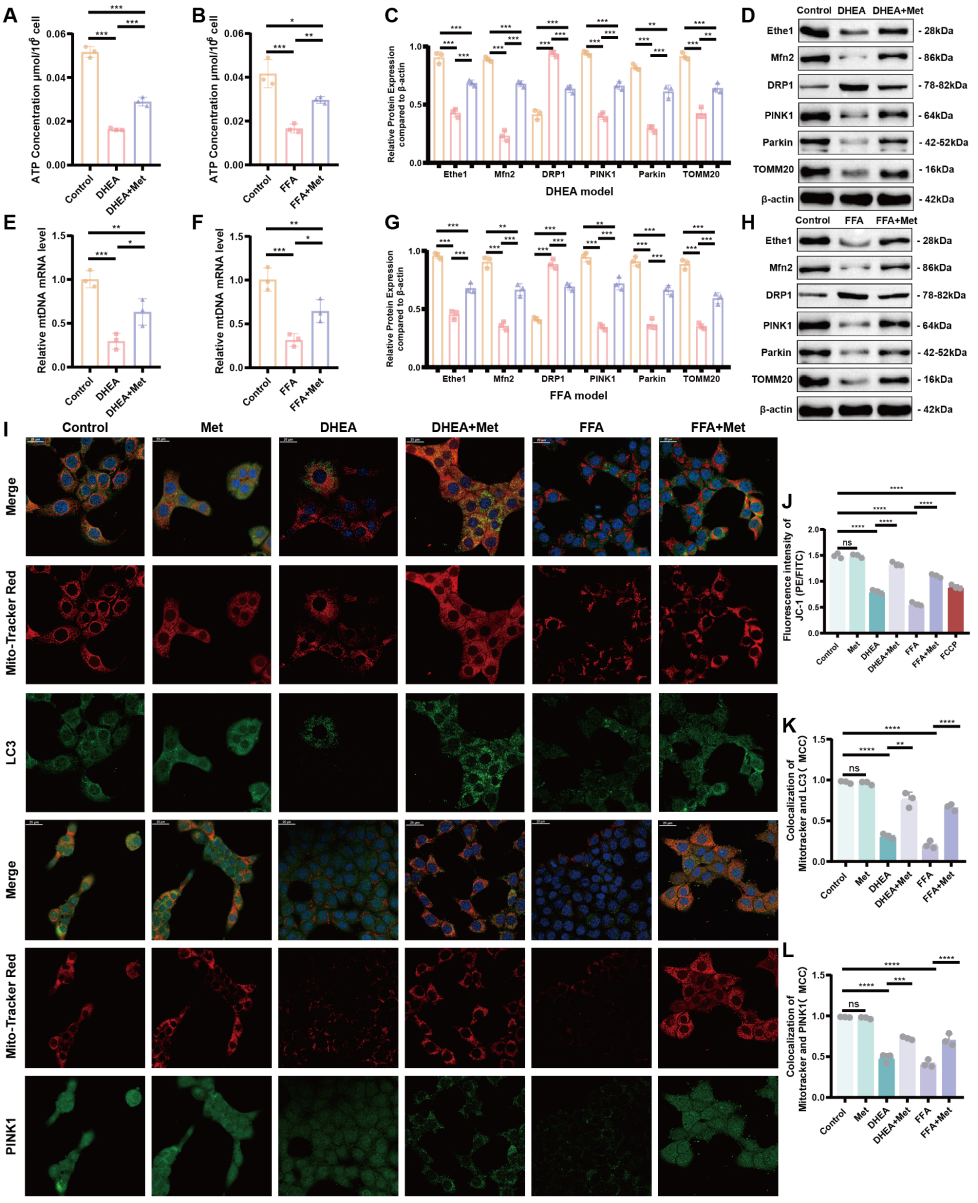
** Metformin improves mitophagy in damaged hepatocytes. (A, B)** Intracellular ATP levels of control (orange bars), DHEA- and FFA-induced (pink bars), and Met-treated (purple bars) cells. **(C, D, G, H)** Expression of Ethe1 and key molecules related to mitochondrial autophagy (Mfn2, DRP1, PINK1, Parkin, and TOMM20) in each group (colors as described for **A** and **B**). **(F, F)** MtDNA copy number for each group. **(I, K, L)** Co-localization of mitochondria with LC3 and Mito-Tracker Red, PINK1, and Mito-Tracker Red under confocal microscopy in the control, DHEA- and FFA-induced, and Met-added cells. **(I)** Mander's co-localization coefficients **(K, L)**. **(J)** Mean fluorescence intensity of JC-1 in each group. All error bars are mean values ± SD, *p-*values were determined by unpaired two-tailed Student's t test (*n* = 3) in independent biological experiments. *p < 0.05; **p < 0.01; ***p < 0.001; ****p < 0.0001.

Metformin (2 μM) was found to significantly reduce the intracellular triglyceride (TG) level and appropriately increase cell activity in the DHEA model (AML-12 cells; Figure 5A, B). A metformin concentration of 10 μM maximized the recovery of cell activity and reduced TG levels in FFA cells (Figure 5C, D). Representative results of Oil Red O staining and intracellular TG detection in DHEA- and FFAs-induced hepatocyte steatosis models are shown in Figure 5F, G, and Figure S2L, respectively. The oxidative stress levels of the cells were determined by measuring the levels of MDA, SOD, GSH, and GSH/GSSG, which were similar to those in the liver tissues of PCOS mice (Figure 5H-L; Figure S2 M-Q). The level of oxidative stress* in vitro* was determined by measuring mitochondrial superoxide. The contents of MitoSOX^TM^ Red and DCFH-DA increased in the DHEA and FFAs groups; however, metformin partially rescued the increase in fluorescence intensity (Figure 5M; Figure S2J, K). Mitochondrial function and the level of mitochondrial autophagy based on intracellular ATP content, mtDNA copy number, protein expression of key molecules related to mitochondrial autophagy (Ethe1, Mfn2, DRP1, PINK1, Parkin, and TOMM20), and mitochondrial membrane potential in DHEA- and FFA-induced cells were evaluated as shown in Figure 6A-L and Figure S2R. The detailed results are presented in the Supplementary Information. The results revealed that the cell model induced by DHEA and FFAs in AML-12 cells had a similar status to those in the PCOS animal model, such as hepatocyte fat deposition, oxidative stress, mitochondrial dysfunction, and weakened mitophagy, while the application of metformin could improve the phenotype to some degree.

**Figure 7:**
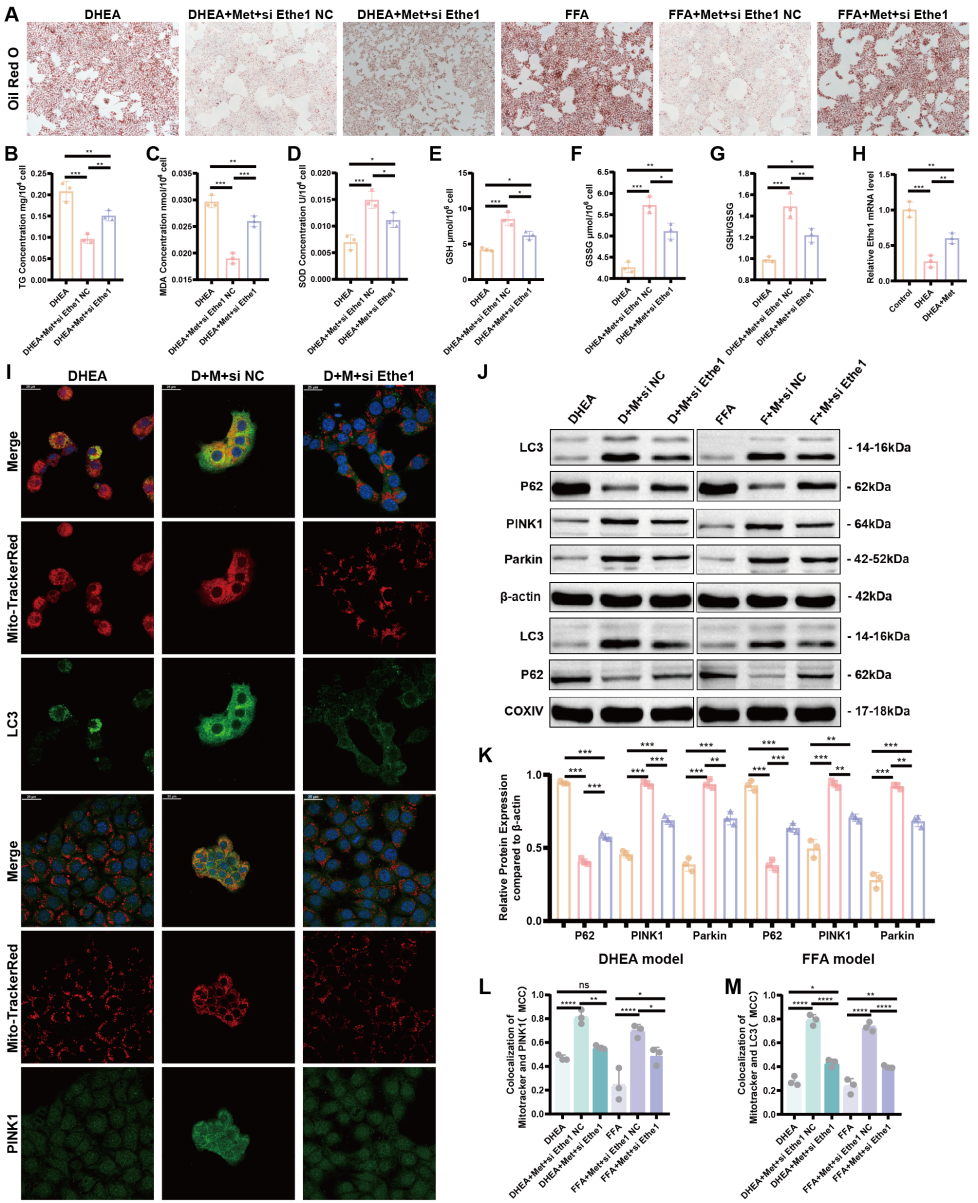
** Metformin reduces DHEA- and FFA-induced hepatocyte injury, alleviates lipid deposition, and improves the mitophagy by activating Ethe1. (A)** Representative images of Oil Red O staining in DHEA/FFA-induced, si-Ethe1 transfected, and Met treated cells. **(B-G)** TG **(B)**, MDA **(C)**, SOD **(D)**, GSH **(E)**, GSSG **(F)**, and GSH/GSSG **(G)** concentration in each group of cells. **(H)** Relative Ethe1 gene expression levels in the control, DHEA-induced, and Met-treated cells. **(I, L, M)** Co-localization of LC3 and PINK1 with Mito-Tracker Red under confocal microscopy in each group of cells. **(I)** Mander's co-localization coefficients **(L, M)**. **(J, K)** Expression of markers related to mitochondrial autophagy (LC3, P62, PINK1, and Parkin) of total protein/mitochondrial protein extractions **(J)** with corresponding gray value levels for the DHEA/FFAs group (orange bars), DHEA/FFAs+Met+si NC group (pink bars), and DHEA/FFAs+Met+si Ethe1 group (purple bars) **(K)**. All error bars are mean values ± SD, *p-*values were determined by unpaired two-tailed Student's t test (*n* = 3) in independent biological experiments. *p < 0.05; **p < 0.01; ***p < 0.001; ****p < 0.0001.

**Figure 8:**
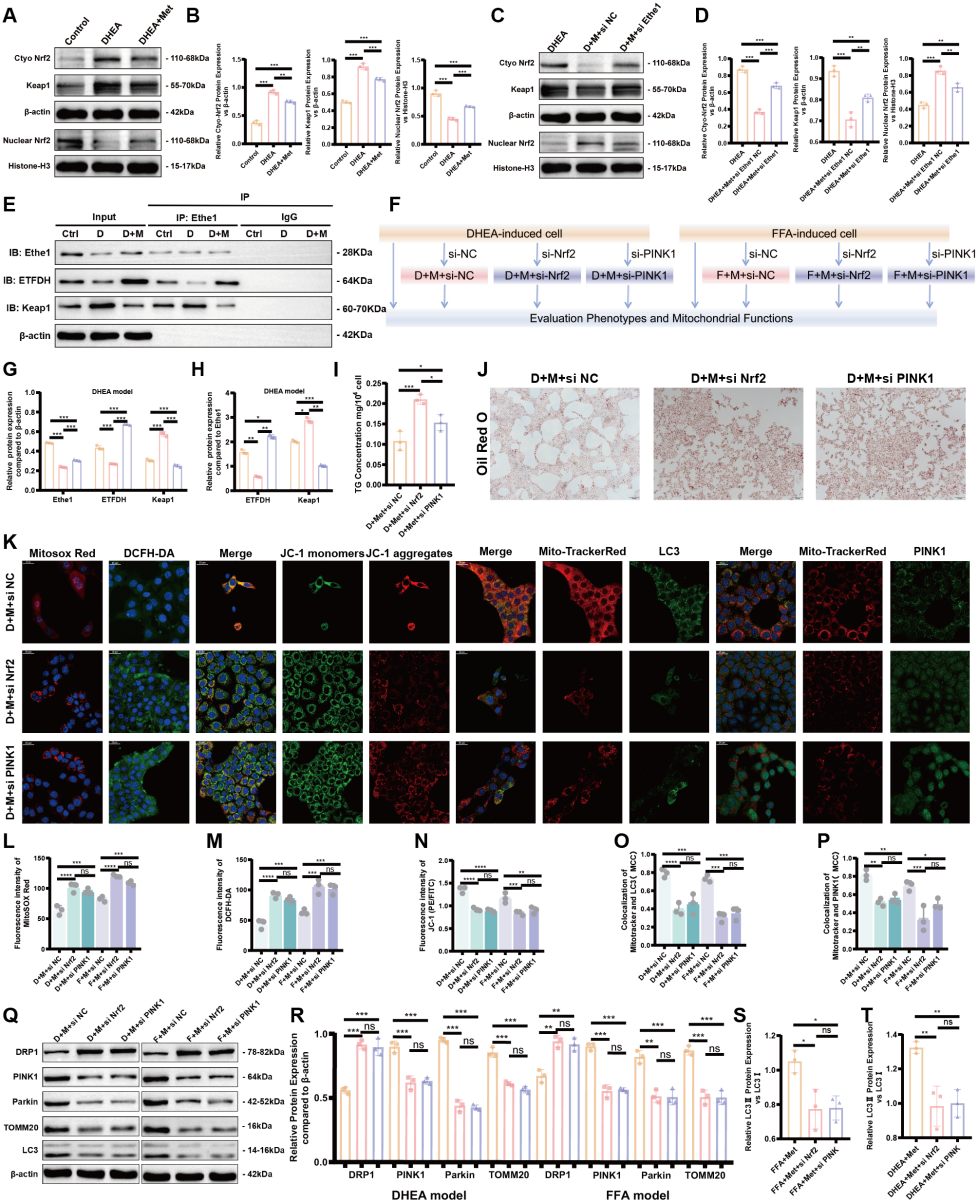
** Metformin alleviates FFA- and DHEA-induced hepatocyte injury by regulating the Ethe1/Keap1/PINK1 pathway through ETFDH. (A, B)** Expression of Keap1 in cells and Nrf2 in the nucleus and cytoplasm of control, DHEA-induced, and Met-treated cells **(A)**. Gray value levels **(B)**. **(C-D)** Expression of Keap1 in cells and Nrf2 in the nucleus and cytoplasm from the DHEA-induced, si-Ethe1, and Met-treated cells **(C)**. Relative gray value levels **(D)**. **(F)** Experimental design flowchart for Nrf2 and PINK1 functions. **(E, G, H)** CO-IP experiments verified the interaction between Ethe1 and ETFDH as well as the interaction between Ethe1 and Keap1 **(E)** in control group (orange bars), DHEA group (pink bars), and DHEA+Met group (purple group) **(G, H)**. **(I, J)** TG concentrations **(I)**. Representative Oil Red O staining imagines for each group **(J)**. **(K, L-P)** Each group of cells was labeled with Mitosox^TM^ Red, DCFH-DA, and JC-1 probes, and the co-localization of LC3 and PINK1 with Mito-Tracker Red in cells from different groups was observed under a confocal microscope **(K)**. Mean fluorescence intensity of Mitosox^TM^ Red, DCFH-DA, and JC-1 **(L-N)**. Mander's co-localization coefficients **(O, P)**. **(Q-T)** Expression of molecules related to mitochondrial autophagy (LC3, DRP1, PINK1, Parkin, and TOMM20)** (Q)** in the DHEA/FFAs+Met+si NC group (orange bars), DHEA/FFAs+Met+si Nrf2 group (pink bars), and DHEA/FFAs+Met+si PINK1 group (purple bars) **(R-T)**. All error bars are mean values ± SD, *p-*values were determined by unpaired two-tailed Student's t test (*n* = 3) in independent biological experiments. *p < 0.05; **p < 0.01; ***p < 0.001; ****p < 0.0001.

We further determined the gene expression levels of Ethe1 in DHEA- and FFA-induced cells, showing that Ethe1 expression was significantly decreased in the model group and increased in the metformin intervention group (Figure 7H; Figure S3 K). We also verified the expression of Keap1 in cells and Nrf2 in the nucleus and cytoplasm. In the DHEA/FFAs model group, Keap1 and Nrf2 expressions increased in the cytoplasm, whereas Nrf2 expression decreased in the nucleus. After metformin treatment, Keap1 and Nrf2 expression increased in the nucleus (Figure 8A, B; Figure S4B, D). These results indicate that the changes in the expression patterns of key molecules (Ethe1, Mfn2, DRP1, PINK1, Parkin, TOMM20, cytoplasmic-Nrf2, nuclear-Nrf2, and Keap1) upon metformin treatment in cell models could mimic those observed in liver samples from the mouse model. Metformin alleviated DHEA- and FFA-induced liver cell injury by regulating mitochondrial autophagy markers.

### Metformin reduces DHEA- and FFA-induced hepatocyte injury, alleviates lipid deposition, and improves mitophagy by activating Ethe1

To elucidate the role of metformin-activated Ethe1 in improving mitochondrial autophagy in damaged hepatocytes, we transfected si-Ethe1 into cells to mimic the reduced expression of Ethe1 in the liver tissue of PCOS mice. Representative Oil Red O staining images are shown in Figure 7A. By measuring the intro-cellular TG content, we found that a high level of lipid deposition remained in cells with Ethe1 knockdown, even after metformin treatment (Figure 7B; Figure S3E). The results for oxidative stress-related indicators (MDA, SOD, GSH, and GSH/GSSG) are shown in Figure 7C-G and Figure S3F-J. In addition, the protein expression of the major molecules of mitochondrial autophagy (LC3, PINK1, and Parkin; Figure 7J, K, and Figure S3L-Q) and co-localization of the mitochondria with LC3 and PINK1 in cells (Figure 7I, L, M; Figure S4A) displayed a similar tendency to PAMH mice liver cells; mitophagy decreased after Ethe1 knockdown, even with metformin treatment. The levels of relevant indicators of mitochondrial function, such as ROS levels within the mitochondria, the overall ROS level in the cell, and mitochondrial membrane potential, are shown in Figure S3A-D. These experimental results indicate that metformin rescued the mitochondrial dysfunction induced by DHEA and FFAs in hepatocytes, which was attenuated by si-Ethe1. Thus, the activation of Ethe1 plays an important role in the metformin medical process. Consequently, Ethe1 knockdown significantly weakens the therapeutic effects of metformin.

### Metformin alleviates FFA- and DHEA-induced hepatocyte injury by regulating the Ethe1/Keap1/Nrf2 pathway through ETFDH

The mechanism by which metformin regulates Ethe1 to improve mitochondrial autophagy has not been previously determined. We hypothesized that metformin regulates Ethe1 through ETFDH, and Ethe1 regulates mitophagy by affecting Keap1/Nrf2 pathway. Thus, we verified the interaction between Ethe1 and ETFDH, and between Ethe1 and Keap1 (Figure 8E, G, H; Figure S4F, G), and measured the expression of Keap1 and Nrf2 in si-Ethe1 cells. The expression of Keap1 and cytosolic Nrf2 were found to be higher in Model+Met+si-Ethe1 cells than in Model+Met+si-NC cells (negative control), whereas that of nuclear Nrf2 was lower, indicating that Ethe1 knockdown reduced Keap1 and Nrf2 uncoupling and inhibited Nrf2 migration from the cytosol to the nucleus, thereby reducing the transmission and activation of mitophagy by Nrf2 in the nucleus (Figure 8C, D; Figure S4C, E). These results suggest that metformin activates Ethe1 expression through ETFDH and exerts its regulatory effect via the Keap1/Nrf2 pathway.

### Metformin alleviates mitochondrial dysfunction by activating the Ethe1/Keap1/Nrf2 pathway and regulating the expression of downstream PINK1/Parkin

PINK1-Parkin is a pivotal molecular pathway that regulates mitophagy. According to previous studies, Nrf2 activates the expression of PINK1-Parkin, thereby promoting mitophagy. To verify whether this phenomenon also occurs in PCOS, we transfected si-Nrf2 and si-PINK1 into DHEA- and FFA-induced model cells after metformin treatment and compared the results to those of the Model+Metformin group to evaluate mitophagy, oxidative stress, lipid deposition levels, and other factors. The experimental design flowchart is shown in Figure 8F.

After the knockdown of Nrf2 and PINK1, the oxidative stress level, mitochondrial function, and mitophagy of cells were decreased even with metformin, as shown by the increased oxidative stress levels, decreased mitochondrial membrane potential, and reduced mitophagy (Figure 8K-P; Figure S5A, B). Figures 8J and S4H show representative images of Oil Red O staining; Nrf2 and PINK1 knockdown led to lipid deposition and increased TG levels in the cells (Figure 8I; Figure S4I). As shown in Figure 8Q-T, the expression of mitophagy-related molecules, such as TOMM20, LC3, PINK1, and Parkin, decreased, while that of the mitochondrial fission protein, DRP1, increased.

Based on these results, after cellular knockdown of Nrf2, the reduced expression of mitophagy proteins could not be rescued by metformin. Similar results also occurred for si-PINK1. Thus, metformin enhances mitochondrial autophagy and improves mitochondrial function, thereby reducing cellular oxidative stress and lipid deposition by activating Ethe1, Keap1/Nrf2, and PINK1/Parkin.

### Mitophagy-related protein levels in PCOS and control women

To investigate whether our findings in PCOS mice are conserved in human PCOS patients, we randomly recruited three PCOS patients. These patients volunteered to participate in the project during outpatient visits, following ethical approval. The same number of control volunteers, with comparable ages, were also randomly recruited. The PCOS patients experienced 10 weeks of metformin treatment and diet control under the guidance of the same dietitian after initial evaluation. Whole-blood samples were collected from the PCOS patients (both before and after metformin treatment) and control volunteers for subsequent biochemical analysis (Table 3).

Compared to control group, women in the PCOS group exhibited a much higher body mass index (BMI) (*p* < 0.05), liver injury, and metabolism levels. Increases in the serum concentrations of AST (*p* < 0.05) and ALT (*p* < 0.01) were observed in women with PCOS. In addition, higher homeostasis model assessment of IR (HOMA-IR) (*p* < 0.01) and fasting insulin levels (FINS) (*p* < 0.05) were observed in the patients with PCOS. An amelioration of AST, ALT, HOMA-IR, FINS, and BMI levels was found in the PCOS patients undergoing metformin treatment and diet control.

The mitophagy-related protein levels in the blood samples were also examined (Figure S6C, D). The levels of these proteins were significantly different between control and PCOS samples. The protein levels of Ethe1, Nrf2, PINK1, and Parkin declined in the PCOS group compared with those in the control group, whereas the protein level of Keap1 increased in the PCOS group. Notably, metformin treatment significantly restored the levels of these mitophagy-related proteins in the PCOS patients. These results are consistent with the observations of the PCOS mice model. Collectively, these data show that in both mice model and human patients, metformin can alleviate liver metabolic dysfunction in PCOS by activating the Ethe1/Keap1/PINK1 pathway.

## Discussion

PCOS is highly associated with NAFLD/MAFLD, as both diseases share common risk factors 36. The mechanisms underlying PCOS and fatty liver comorbidity are mainly attributed to insulin resistance and hyperandrogenism and are associated with abnormal glycometabolism, dyslipidemia, obesity, and chronic inflammation 37. The synergy between these conditions extends beyond their mutual pathophysiology and development, with their co-existence accelerating the rate and risk of complications, leading to a higher prevalence of adverse cardiovascular and other metabolic and neoplastic outcomes than observed with either condition alone 2. At PCOS diagnosis, screening for MAFLD is necessary because most patients with MAFLD are asymptomatic. Early detection of MAFLD in patients with PCOS is important because timely intervention in patients with steatosis or steatohepatitis can reduce the probability of liver disease progression 36.

Mitophagy is one of the important mechanisms for regulating lipid metabolism in liver steatosis. Tao-Hong-Si-Wu-Tang was found to profoundly repair lipid metabolism dysfunction and liver fibrosis by promoting mitophagy 38. Tectorigenin decreased the expression of tRF-3040b to enhance mitophagy and reduce lipid deposition, inflammation, and pyroptosis, thereby inhibiting pyroptosis in metabolic dysfunction-associated steatohepatitis 39. Magnoflorine was found to promote Parkin/PINK1-mediated mitochondrial autophagy, thereby inhibiting NLRP3/Caspase-1-mediated pyroptosis, ultimately decreasing tubular cell steatosis, lipid deposition, tubular dilation, and glomerular fibrosis in chronic kidney disease 40. These findings suggest that promoting mitophagy is an effective approach to reduce lipid deposition, the specific mechanisms of which remain to be investigated.

Metformin affects the ATP/AMP ratio and activates AMPK, which subsequently regulates lipid metabolism. A recent study revealed that high-dose metformin (over 250 mg/kg) can activate AMPK by inhibiting the mitochondrial complex 1 41, whereas low-dose metformin (5 uM) targets the lysosomal AMPK pathway to decrease hepatic triglyceride levels through the PEN2-ATP6AP1 axis in an AMP-independent manner 42. A moderate dose of metformin enables the alleviation of NAFLD and cirrhosis, liver damage relief, and reduced incidence of hepatocellular carcinoma and cholangiocarcinoma 41. Such findings reflect the dose-dependent effects of metformin, which should be considered when determining the applicable dose in the context of different disease models. Previous studies have explored the optimal therapeutic dose of metformin in type 2 diabetes animal models and determined that a therapeutic concentration of 50 mg/kg achieved comparable plasma metformin concentrations as observed in patients with metformin-treated type 2 diabetes 42,43. Meanwhile, many studies have applied various doses of metformin in different PCOS animal models; however, therapeutic doses of metformin in PAMH-induced PCOS models have remains unexplored 32,42-53. The results of our study verified that a moderate metformin dose (50 mg/kg/day) for 2 months was more effective than a high (250 mg/kg/day) or low (10 mg/kg/day) dose at reducing the amount of triglycerides and mitochondrial damage in the liver. Thus, for the first time, we identified that 50 mg/kg is the optimal medical treatment dosage among the three doses for PAMH-induced PCOS-like mice.

Previous studies have revealed that metformin can protect against chemical- and drug-induced liver injury caused by hepatotoxic drugs. Metformin also reduces ROS production by inhibiting mitochondrial complex 1, which reduces liver damage. These findings suggest that metformin has pharmacological effects against liver diseases 26. However, research on the role of metformin in MAFLD/NAFLD related to PCOS had remained insufficient. In our study, we revealed that metformin treatment can also mitigate the abnormal metabolism in MAFLD/NAFLD related to PCOS.

At a molecular level, metformin regulates mitophagy through the Keap1/Nrf2 pathway, which is an important defense signal-transduction pathway in the body. Nrf2 can regulate most autophagy-related proteins through Keap1 54. As an autophagy adaptor protein, p62 links autophagy to the Keap1/Nrf2 pathway 55; p62 can competitively bind to Keap1 and sequester Keap1 into autophagosomes, which impairs the ubiquitylation of Nrf2. This leads to the activation of the Nrf2 signaling pathway and the expression of its downstream genes 56. The binding of Nrf2 to the PINK1 promoter can activate the transcription of PINK1, ultimately enhancing mitophagy 20.

Previous research has indicated that in some disease models, such as brain ischemia/reperfusion, cardiac injury, chronic kidney disease, and type 2 diabetes models, mitochondrial damage and some other disease phenotypes were rescued by metformin treatment through the regulation of the above-mentioned mitophage-related molecules. However, whether metformin can mitigate abnormal metabolism in MAFLD/NAFLD related to PCOS through mitophagy regulation remained unclear. In this study, we discovered that metformin treatment restored the mitophage defect in the liver cells of the PAMH-induced PCOS model through the Keap1/Nrf2 pathway.

It was unclear how metformin directly or indirectly regulates the Keap1/PINK1 pathway to enhance mitochondrial autophagy. To elucidate the specific regulatory mechanism of metformin in PCOS-related liver damage, we investigated the pathways and molecules regulated by metformin and subsequently linked these to the key factors associated with MAFLD related to PCOS. The targets of metformin were obtained from DrugBank and genome-wide association study data from the MRC Integrative Epidemiology Unit (IEU) OpenGWAS and FinnGen. These targets were further used to identify potential mechanisms through which metformin alleviates metabolism defects in MAFLD/NAFLD related to PCOS. Notably, ETFDH is one of the 10 primary targets of metformin 57,58. Our results revealed an interactive relationship between ETFDH and Ethe1. Ethe1 was downregulated in the PAMH-induced PCOS group but upregulated upon metformin treatment. This suggests that metformin may mediate Ethe1 up-regulation through ETFDH.

Ethe1 encodes a member of the metallo beta-lactamase family of iron-containing proteins involved in the mitochondrial sulfide oxidation pathway; the encoded protein catalyzes the oxidation of a persulfide substrate to sulfite 59. Ethe1 was first reported in ethylmalonic encephalopathy, where mutations in this gene lead to metabolic disorders in the gastrointestinal tract and peripheral blood vessels. According to recent studies, this gene is associated with liver injury 60 and mitochondrial dysfunction 61,62. A previous study confirmed that uranium depletion causes oxidative stress and antioxidant defense imbalance in renal cells by down-regulating the ETHE1/Nrf2 pathway, which further leads to mitochondrial dysfunction and ultimately, nephrotoxicity 27. Another study found that liver-targeted adeno-associated virus-mediated ETHE1 gene transfer markedly improved both the clinical course and metabolic abnormalities in mice with ethylmalonic encephalopathy 63. Such finding suggests that the liver is one of the most important organs for regulating the expression and function of Ethe1. Our results confirm the interaction between Ethe1 and Keap1, and the role of Ethe1 in Nrf2 entry into the nucleus by regulating the uncoupling of Keap1 and Nrf2. In addition, for the first time, we identified Ethe1 as a key molecule linking ETFDH and Keap1 and illustrated the regulatory effect of metformin on the Keap1/PINK1 pathway through Ethe1 in the liver of the PCOS model.

We could not identify the specific mechanism whereby metformin acts on ETFDH and functions, which served as the main limitation of this study. Whether metformin uses other pathways to regulate Ethe1 other than through ETFDH remains to be elucidated. Furthermore, we did not clearly define whether the interaction between ETFDH and Ethe1 is one-to-one or one-to-more. Similarly, we only verified the interaction between Ethe1 and Keap1, whereas Ethe1 might regulate Nrf2 via other pathways. The therapeutic effects of metformin on NAFLD-related liver injury are possibly due, at least in part, to metabolic improvement through signaling pathways beyond the Ethe1/Keap1/Nrf2/PINK1/Parkin pathway. This requires further investigation.

We speculate that ETFDH is one of the main targets of metformin, possibly through its up-regulation of this target, which activates Ethe1 expression and ultimately alleviates mitochondrial dysfunction, especially mitophagy, leading to lipid metabolic reduction in the liver. The molecular mechanisms by which metformin regulates mitochondrial function and lipid metabolism by down-regulating Ethe1 will be explored in the future.

## Conclusion

In this study, we demonstrated that metformin alleviates PAMH-induced MAFLD by modulating mitochondrial function and redox homeostasis, particularly mitophagy, mainly via the Ethe1/Keap1/Nrf2/PINK1/Parkin pathway. Our results highlight a novel mechanism whereby metformin reduces hepatic lipid deposition. As metformin is a widely used clinical drug with relatively low toxicity and few side effects, our findings provide compelling evidence supporting its potential therapeutic application in the prevention of NAFLD/MAFLD in PCOS.

## Supplementary Material

Supplementary figures and tables.

## Figures and Tables

**Table 1:** Sequences of Ethe1, Nrf2, and PINK1 siRNA

ID	Sequence information (5'-3')
siRNA Ethe1	CGGGAUGCUCAGUUGAUUA(dT)(dT)
	UAAUCAACUGAGCAUCCCG(dT)(dT)
siRNA Nrf2	CCGAAUUACAGUGUCUUAA(dT)(dT)
	UUAAGACACUGUAAUUCGG(dT)(dT)
siRNA PINK1	CUGAAAUUGGACAAGAUGA(dT)(dT)
	UCAUCUUGUCCAAUUUCAG(dT)(dT)

**Table 2:** Sequences of primers for PCR

Gene	PCR primer sequences
*Mus actin_F*	GTCCCTCACCCTCCCAAAAG
*Mus actin_R*	GCTGCCTCAACACCTCAACCC
*Mus mtDNA_F*	GCCGGTGACTACGACTGAA
*Mus mtDNA_R*	CACTGGCCTGCAAGTCTTC
*Mus Ethe1_F*	ACTCACTGCCATGCTGACC
*Mus Ethe1_R*	TCGAGTCTCCAAAGCAAAGC
*Mus Nrf2_F*	CCCAGCACATCCAGACAGA
*Mus Nrf2_R*	CCAGAGAGCTATTGAGGGACTG
*Mus PINK1_F*	AGATGGTCCCAAGCAGCTT
*Mus PINK1_R*	TGCAAGGTCATCATGGTAGC

**Table 3:** Clinical parameters of PCOS and healthy women

Parameters	Control(n=3)	PCOS(n=3)	*p*-value(Control vs PCOS)	PCOS+Met (n=3)	*p*-value(PCOS vs PCOS+Met)
Age(years)	32.00±3.606	30.33±6.658	ns	30.33±6.658	ns
BMI(Kg/m^2^)	23.21±2.051	29.23±1.168	*p <0.05*	25.89±2.303	ns
FINS(uIU/ml)	6.200±0.7550	20.30±7.539	*p <0.05*	8.100±2.848	*p <0.05*
FBG(mmol/l)	4.533±0.4726	5.733±1.629	ns	5.000±1.039	ns
HOMA-IR	1.257±0.2593	4.943±1.205	*p < 0.01*	1.817±0.7431	*p < 0.01*
TC (mmol/L)	3.717±0.4508	5.477±2.245	ns	4.913±1.339	ns
TG (mmol/L)	0.6800±0.2606	1.890±1.142	ns	0.9433±0.4725	ns
HDL-C (mmol/L)	1.323±0.2065	1.137±0.1443	ns	1.243±0.2538	ns
LDL-C (mmol/L)	2.093±0.5620	3.567±1.472	ns	3.060±0.9430	ns
AST U/L	19.67±3.215	30.00±5.000	*p < 0.05*	18.67±2.082	*p < 0.05*
ALT U/L	14.00±4.359	52.00±12.12	*p < 0.01*	18.33±2.309	*p < 0.01*
FSH(IU/l)	7.073±2.060	5.620±2.004	ns	3.220±2.713	ns
LH(IU/l)	3.690±0.5237	6.437±1.721	ns	3.680±1.908	ns
LH/FSH	0.5467±0.1582	1.257±0.5178	ns	1.670±1.022	ns
Estradiol (pg/mL)	31.33±3.055	51.33±15.82	ns	136.7±82.59	ns
Progesterone (ng/mL)	0.9233±0.5499	0.2767±0.2401	ns	3.560±5.277	ns
Testosterone (ng/mL)	0.2700±0.02646	0.7067±0.3050	ns	0.4467±0.1930	ns

p-values > 0.05 were considered non-significant (ns). BMI, Body mass index; HOMA-IR=Fasting Glucose (FGB)(mmol/l) * Fasting insulin (FINS)(uIU/mL)/22.5; TC, total cholesterol; TG, triglyceride; HDL, high-density lipoprotein; LDL, low-density lipoprotein; LH, luteinizing hormone; FSH, follicle-stimulating hormone.

## Abbreviations

PCOS: Polycystic ovary syndrome
NAFLD: Non-alcoholic fatty liver disease
MAFLD: Metabolic dysfunction-associated fatty liver disease
ROS: Reactive oxygen species
MDA: Malondialdehyde
NFE2L2: NFE2 like bZIP transcription factor 2
KEAP1: Kelch-like epichlorohydrin-associated protein 1
PAMH: Prenatal anti-Müllerian hormone
PBS: Phosphate-buffered saline
OGTT: Oral glucose tolerance test
ITT: Insulin tolerance test
NCD: Normal chow diet
VO_2_: Rate of oxygen consumption
VCO_2_: Rate of carbon dioxide production
RER: Respiratory exchange ratio
SOD: Superoxide dismutase
GSH: Glutathione
GSSG: Glutathione oxidized
AML-12: Alpha mouse liver 12
Co-IP: Co-immunoprecipitation
SDS-PAGE: Sodium dodecyl sulfate-polyacrylamide gel electrophoresis
ANOVA: Analysis of variance
AUC: Area under the curve
DRP1: Dynamin-related protein 1
VDAC1: Voltage-dependent anion channel 1
ETFDH: Electron transfer flavoprotein dehydrogenase
Ethe1: Ethylmalonic encephalopathy protein 1
FFAs: Free fatty acids
TG: Triglyceride
