# Supplementary material for: Metformin Alleviates Liver Metabolic Dysfunction in Polycystic Ovary Syndrome by Activating the Ethe1/Keap1/PINK1 Pathway
Source: Int J Biol Sci. 2025 May 21;21(8):3505–26. doi: 10.7150/ijbs.104778 (PMC12160571; doi:10.7150/ijbs.104778)
Supplement: Supplementary file 1 — Supplementary figures and tables. [file ijbsv21p3505s1.pdf]

Supplementary Materials

Metformin Alleviates Liver Metabolic Dysfunction in Polycystic Ovary Syndrome by Activating the Ethe1/Keap1//PINK1/ Pathway

Short title: Metformin Alleviates PCOS liver mitochondria dysfunction

Yuan Xie[1+], Ying Tian[1+], Junting Huang[1], Wanying Deng[2], Xiaohui Li[1], Yujia Liu[1, 3], Hao liu[4], Lei Gao[5]*, Qiu Xie[6]*, and Qi Yu[1]*

[1]Department of Obstetrics and Gynecology, National Clinical Research Center for Obstetric & Gynecologic Diseases, State Key Laboratory for Complex Severe and Rare Diseases, Peking Union Medical College Hospital, Chinese Academy of Medical Sciences & Peking Union Medical College, Peking Union Medical College Hospital (Dongdan Campus), No.1 Shuaifuyuan Wangfujing Dongcheng District, Beijing, 100730, China.

[2]Center of Reproductive Medicine, Department of Obstetrics and Gynecology, Shengjing Hospital of China Medical University, Shenyang, China.

[3]Eight-year Medical Doctor Program, Chinese Academy of Medical Sciences and Peking Union Medical College, Beijing 100730, China.

[4]Department of Child and Adolescent Health, Public Health College, Harbin Medical University, Harbin 150081, China.

[5]Key Laboratory of Epigenetic Regulation and Intervention, Institute of Biophysics, Chinese Academy of Sciences, Beijing, 100101, China

[6]Department of Medical Research Center, State Key Laboratory for Complex Severe and Rare Diseases, Peking Union Medical College Hospital, Chinese Academy of Medical Science and Peking Union Medical College, Beijing 100730, China.

[+]Contributed equally.
[*]Corresponding author.

1 Supplementary Figures and legends

Figure S1

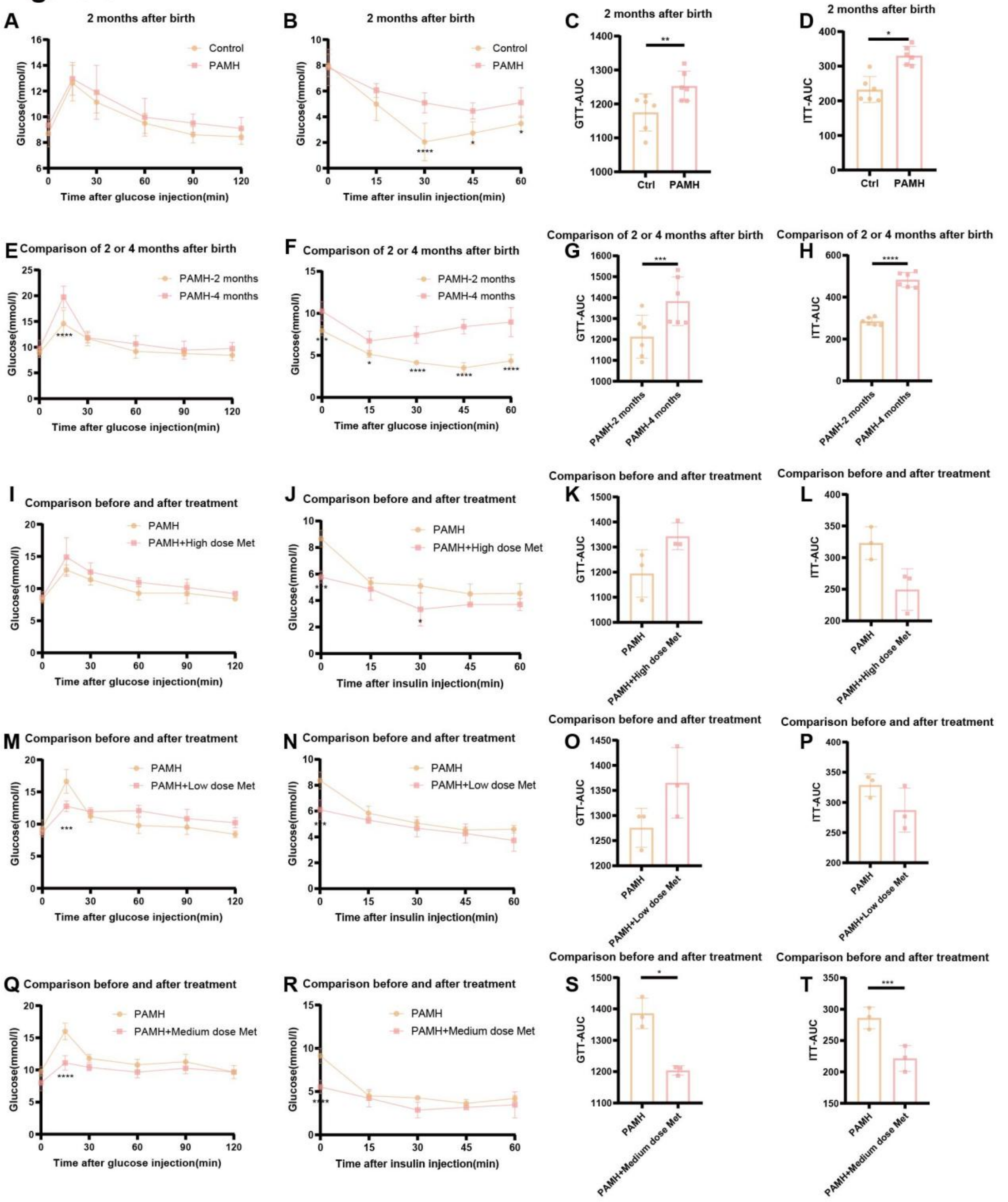

Figure S1 | Metformin treatment alleviated metabolic abnormalities after PAMH induction. (A–D) Comparison of GTT and ITT experiments between PAMH induced and control mice at 2 months after birth **(A, B)**, and a comparison of the AUC (*n* = 6). **(C, D)**. **(E, H)** Comparison of GTT **(E)** and ITT **(F)** results of the same PAMH mice at 2 and 4 months after birth with corresponding AUC values of 4-month-old PAMH mice **(G, H)** (*n* = 6). **(I–T)** Changes in the GTT and ITT experiments and AUC results of the same PAMH-induced mice before and after high-dose metformin **(I–L)**, low-dose Met **(M–P)**, and medium-dose Met **(Q–T)** treatment (*n* = 3). All error bars

are mean values ± SD, p-values were determined by unpaired two-tailed Student's t test ($n = 3$) in independent biological experiments. *$p < 0.05$; **$p < 0.01$; ***$p < 0.001$; ****$p < 0.0001$.

Figure S2

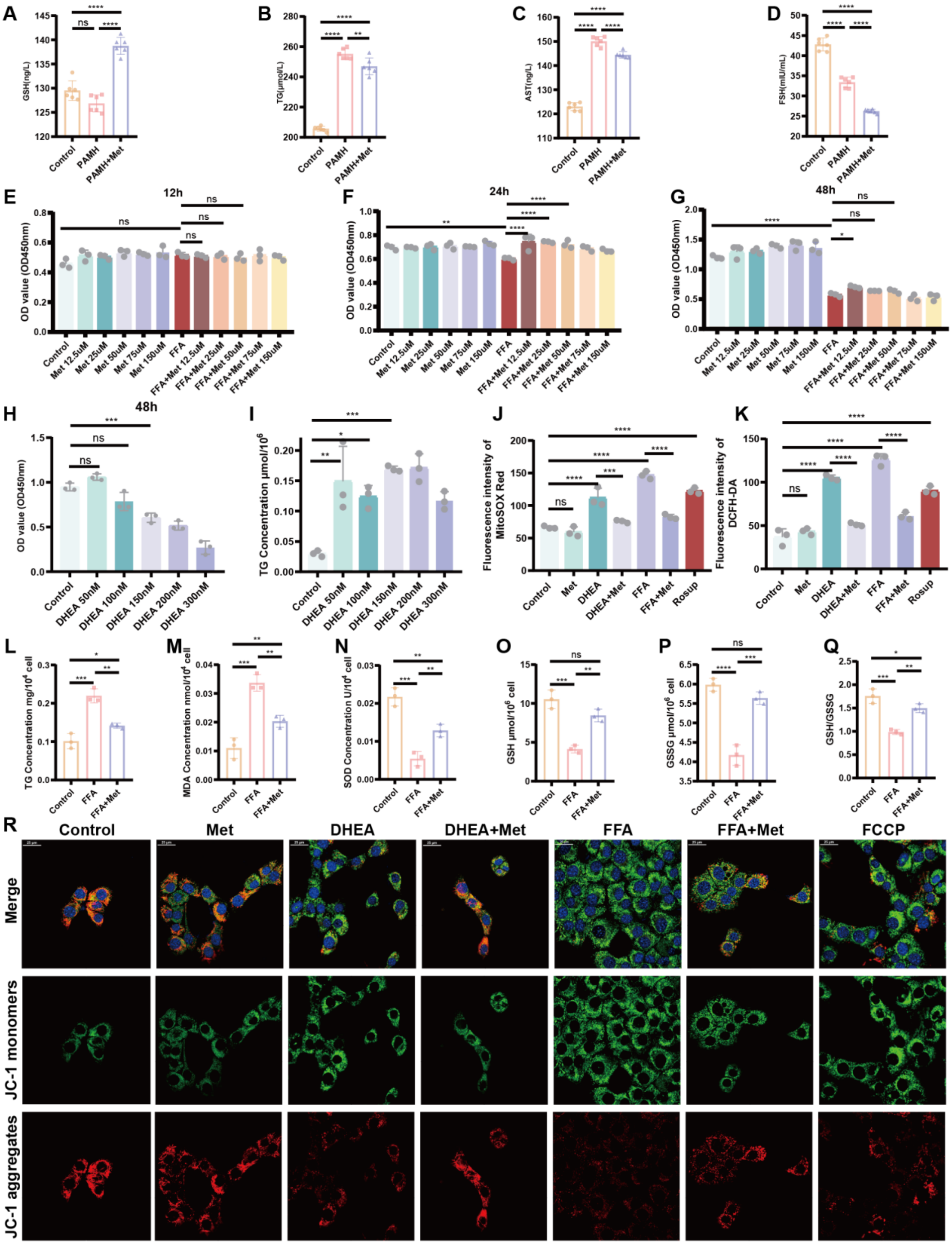

Figure S2 | Metformin alleviated the metabolic abnormalities and mitochondrial dysfunction in the DHEA/FFA-induced cells. (A–D) Serum concentrations of glutathione (GSH) **(A)**, triglyceride (TG) **(B)**, aspartate transaminase (AST) **(C)**, and follicle-stimulating hormone (FSH) **(D)**. **(E–G)** Treatment with Met at five different concentrations (12.5, 25, 50, 75, and 150 μM) at three time points (12 h **(E)**, 24 h **(F)**, and 48 h **(G)**) with CCK8 experiments. **(H, I)** Exploration of a suitable concentration for DHEA to induce AML-12 cell hepatocyte steatosis altering based in CCK8 results **(H)**. TG concentrations **(I)**. **(J, K)** Mean fluorescence intensity of Mitosox^{TM} Red **(J)**

and DCFH-DA **(K)** in the control, DHEA/FFA-induced, and metformin-treated cells. **(L–Q)** TG **(L)**, MDA **(M)**, SOD **(N)**, GSH **(O)**, GSSG **(P)**, and GSH/GSSG **(Q)** concentrations in each FFA-induced and Met-disposed group. **(R)** Cells were labeled with JC-1 probes and observed under confocal microscope for each group. All error bars are mean values ± SD, *p*-values were determined by unpaired two-tailed Student's t test ($n = 3$) in independent biological experiments. *$p < 0.05$; **$p < 0.01$; ***$p < 0.001$; ****$p < 0.0001$.

Figure S3

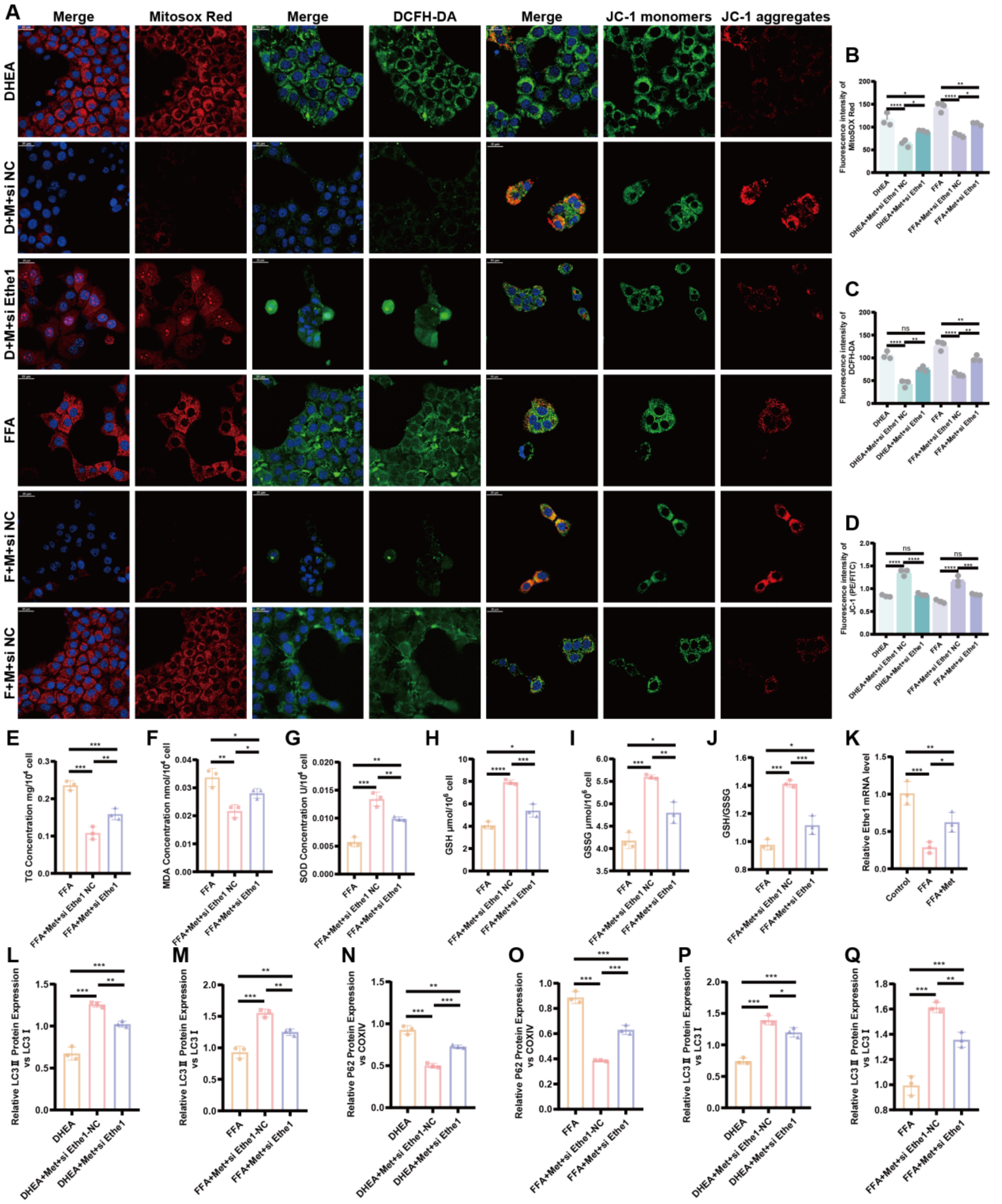

Figure S3 | Metformin reduces DHEA- and FFA-induced mitochondrial dysfunction of AML-12 cells by activating Ethe1. (A–D) Cells were labeled with Mitosox^{TM} Red, DCFH-DA, and JC-1 probes and observed under a confocal microscope in DHEA/FFA-induced cells, si-Ethe1 transfected combine Met treated and DHEA/FFA-induced cells, and si-NC transfected with Met and DHEA/FFA-induced cells **(A)**. **(B–D)** Mean fluorescence intensity. **(E–J)** TG **(E)**, MDA **(F)**, SOD **(G)**, GSH **(H)**, GSSG **(I)**, and GSH/GSSG **(J)** concentrations in each group. **(K)** Relative Ethe1 gene expression level in control, FFA-induced, and Met-treated cells. **(L–Q)** Gray value calculations of relative expression of LC3 and p62 in total protein/mitochondrial proteins, which are not described in Figure 7K. All

error bars are mean values ± SD, *p*-values were determined by unpaired two-tailed Student's t test ($n = 3$) in independent biological experiments. *$p < 0.05$; **$p < 0.01$; ***$p < 0.001$; ****$p < 0.0001$.

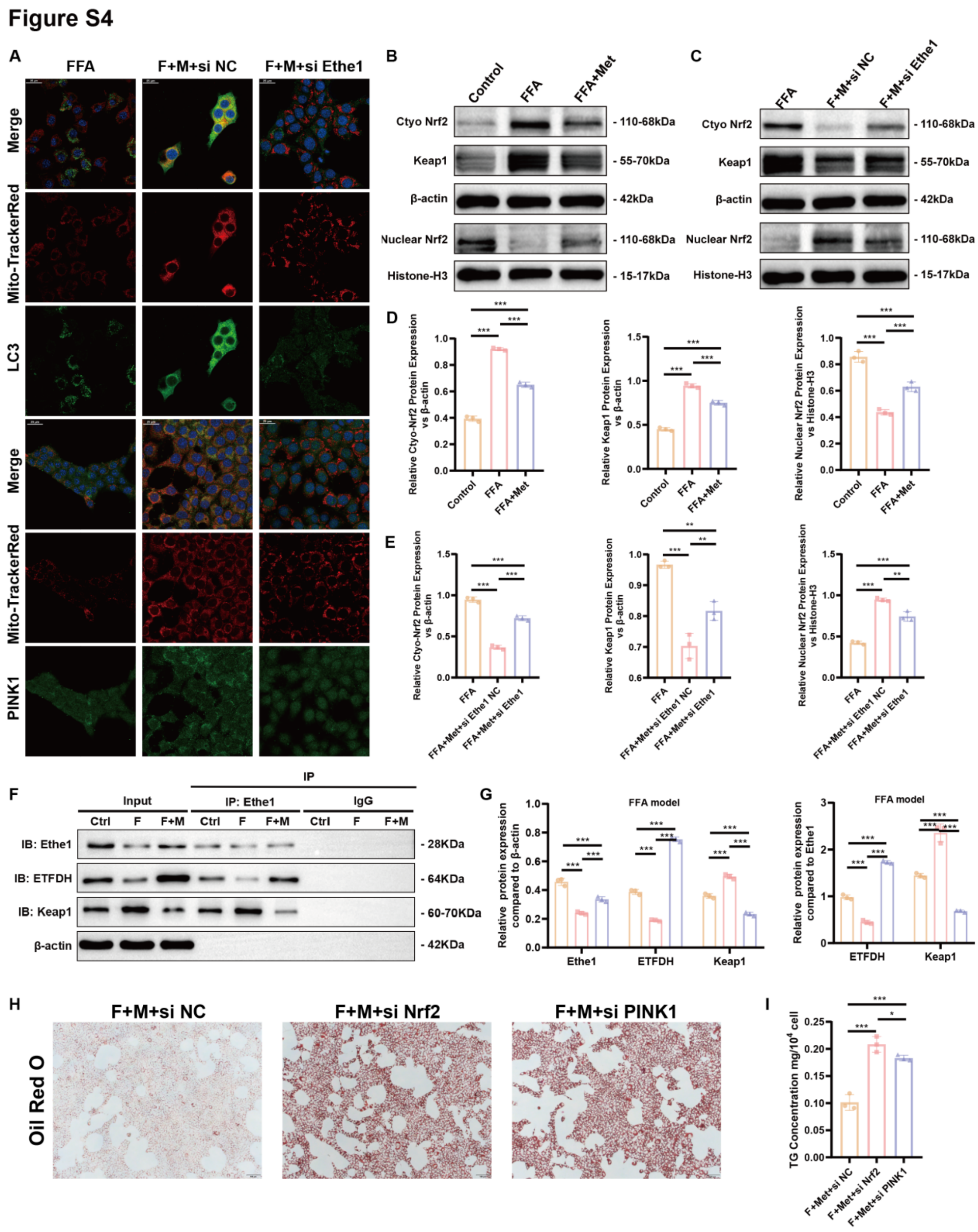

Figure S4 | Metformin alleviates FFA-induced injury by regulating the Ethe1/Keap1/Nrf2/PINK1/Parkin pathway through ETFDH. (A) Co-localization of LC3 and PINK1 with Mito-Tracker Red under confocal microscopy in each group. **(B, D)** Expression of Keap1 in cells and Nrf2 in the nucleus and cytoplasm from the control, FFA-induced, and Met-treated cells **(B)**. Gray value levels **(D)**. **(C, E)** Expression of Keap1 in cells and Nrf2 in the nucleus and cytoplasm from FFA-induced, si-Ethe1, and Met-treated cells **(C)**. Relative gray value levels **(E)**. **(F, G)** CO-IP experiments verified the interaction between Ethe1 and ETFDH, as well as the interaction between Ethe1 and Keap1 in FFA **(F)** induced in each group. Relative expression levels are shown for the control group (orange bars), FFA group (pink

group), and FFA+Met group (purple bars) **(G)**. **(H, I)** Representative images of Oil Red O staining for each group **(H)**. TG concentrations **(I)**. All error bars are mean values ± SD, *p*-values were determined by unpaired two-tailed Student's t test ($n = 3$) in independent biological experiments. *$p < 0.05$; **$p < 0.01$; ***$p < 0.001$; ****$p < 0.0001$.

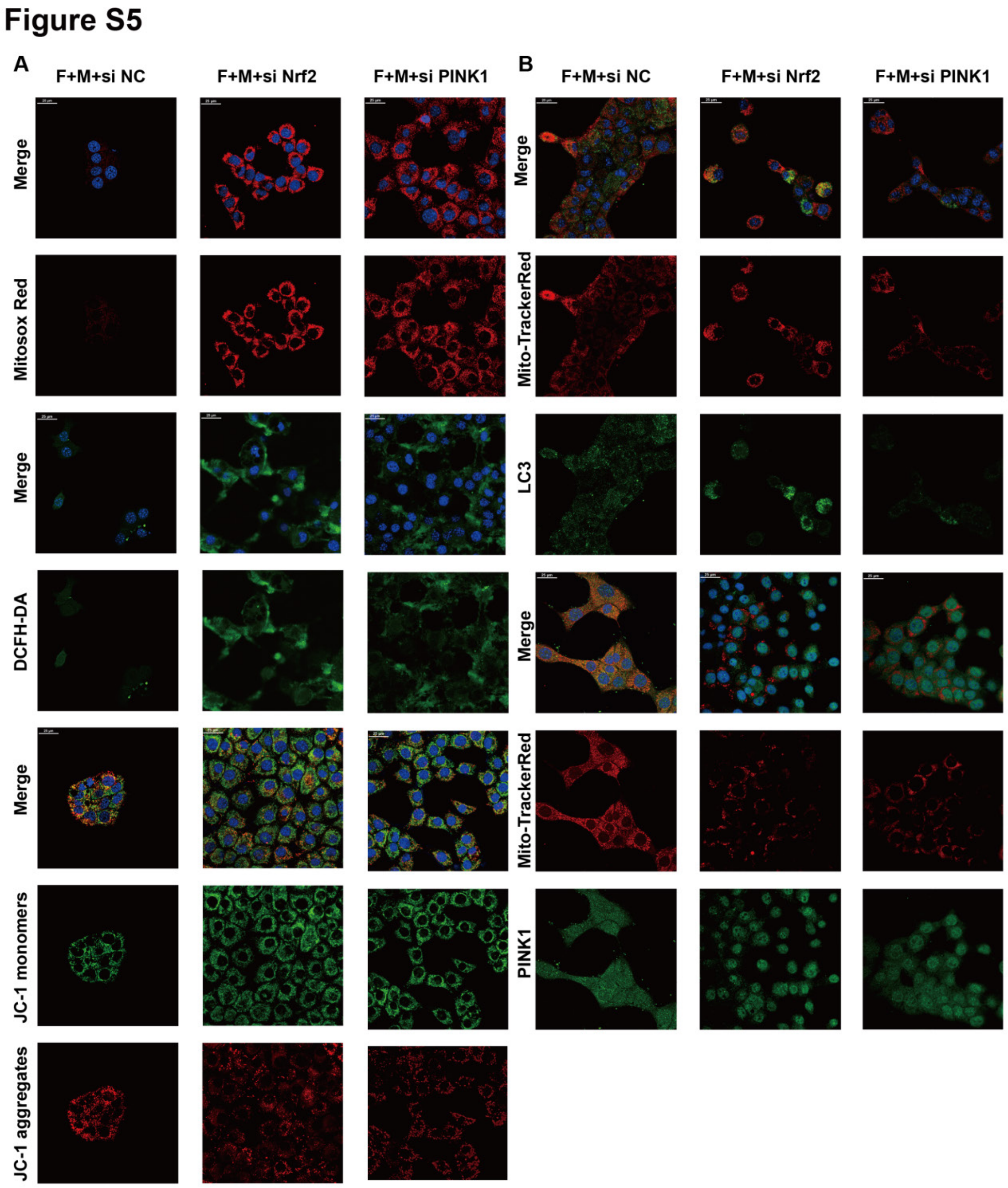

Figure S5 | Through regulating the Ethe1/Keap1/Nrf2/PINK1/Parkin pathway, metformin alleviated FFA-induced mitochondrial dysfunction. (A) Cells were labeled with Mitosox^{TM} Red, DCFH-DA, and JC-1 probes and observed under a confocal microscope in si+NC+Met+FFA cells, si-Nrf2+Met+FFA cells and si-PINK1+Met+FFA cells. **(B)** Co-localization of mitochondria with LC3 and PINK1 and Mito-Tracker Red under confocal microscopy in each group. All error bars are mean values ± SD, *p*-values were determined by unpaired two-tailed Student's t test ($n = 3$) in independent biological experiments. *$p < 0.05$; **$p < 0.01$; ***$p < 0.001$; ****$p < 0.0001$.

Figure S6

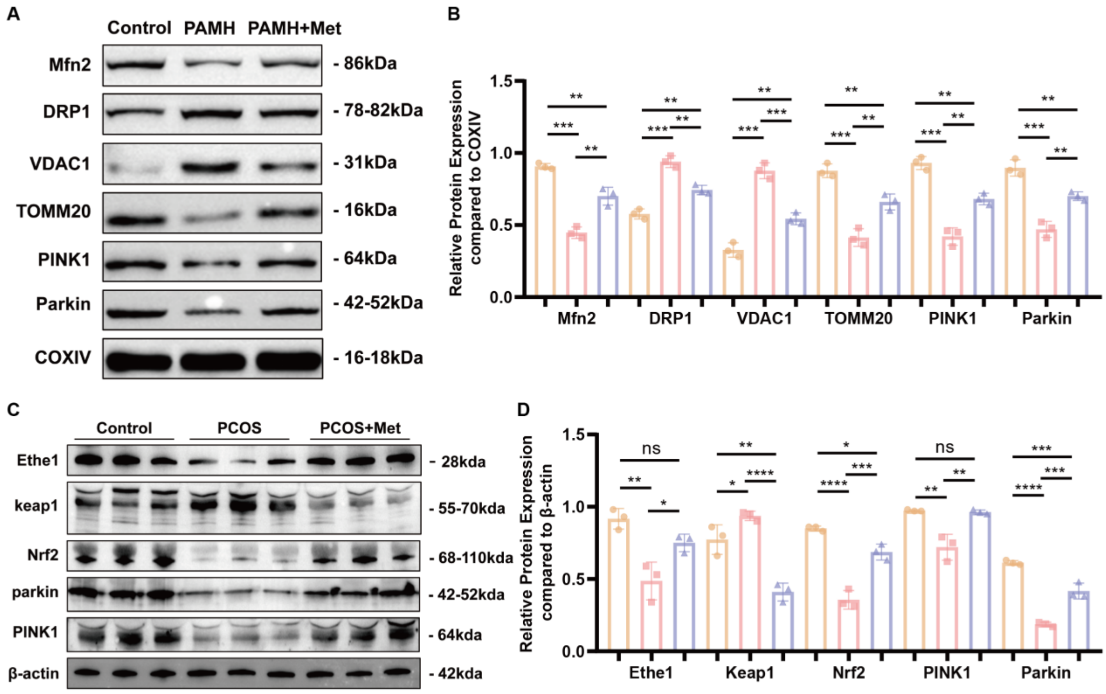

Figure S6 | Mitophagy-related protein levels in PCOS-like mice and PCOS patients. (A, B) Mitochondrial proteins extracted from fresh liver tissues in each group and the expression of the mitophagy-related markers Mfn2, DRP1, VDAC1, TOMM20, PINK1, and Parkin. **(C, D)** Mitophagy-related protein levels in the acquired blood samples and the relative expression levels for the control group (orange bars), PAMH/PCOS group (pink bars), and PAMH/PCOS+Met group (purple bars). All error bars, mean values ± SD, p values were determined by unpaired two-tailed Student's t test of n = 3 independent biological experiments. *$p < 0.05$; **$p < 0.01$; ***$p < 0.001$; ****$p < 0.0001$).

2 Supplementary Methods and Materials

2.1 Cytological Examination of Vaginal Exfoliation

Vaginal smears were conducted over 7 days to monitor changes in the mice estrous cycle. A total of 10–20 μL of physiological saline was injected into the mouse vagina, followed by a swab to collect cells, which were then transferred onto a dry glass slide. The slides were air-dried, and the cells were fixed with methanol for 3 min. After fixation, the morphology of the vaginal cells was observed under a microscope. Wright's–Giemsa staining reagent (G1020, Solarbio, China) was applied, and cell morphology was examined at 10× magnification. The determination of the estrous cycle phases (proestrus, estrus, metestrus, and diestrus) was based on the predominant cell types: nucleated epithelial cells in proestrus, keratinized epithelial cells in estrus, and leukocytes in diestrus.

2.2 Measurement of mice response to insulin and glucose

Female offspring underwent insulin tolerance tests (ITTs) and glucose tolerance tests (GTTs) as previously described [1] at 2 and months after birth, and both before and after metformin treatment.

2.3 Serum analysis of mouse sex hormones

Blood samples were collected from the abdominal aorta and centrifuged at 3500 ×*g*/min for complete serum separation. The serum was then used for various biochemical tests. Levels of sex hormones in the serum were quantified using the following ELISA kits: luteinizing hormone (LH) (E-EL-M3053, Elabscience), follicle-stimulating hormone (FSH) (E-EL-M0511, Elabscience), and testosterone (T) (E-OSEL-M0003, Elabscience). Additionally, biochemical markers including glutamic oxalacetic transaminase (AST) (JM-03113M1), glutamic-pyruvic transaminase (ALT) (JM-03154M1), glutathione (GSH) (JM-03082M1), total cholesterol (TC) (JM-02912M1), triglycerides (TG) (JM-03100M1), high-density

lipoprotein (HDL-C) (JM-03110M1), and low-density lipoprotein (LDL-C) (JM-03080M1) were measured using ELISA kits from Jingmei Biotechnology (Yancheng, Jiangsu, China) according to the manufacturer's instructions.

2.4 Histological assessment of mouse ovaries and livers

Dissected ovaries and liver tissue samples were fixed in 4% paraformaldehyde (P1110, Solarbio, China) overnight at 4 °C. Following dehydration and paraffin embedding, the tissues were sectioned into 4–5-μm slices. Hematoxylin and eosin (H&E) staining (G1120, Solarbio) was performed for histological analysis. The stained ovarian and liver tissues were then examined using the Nano Zoomer Digital Pathology RS system.

2.5 Masson's trichrome staining

Liver paraffin sections were dehydrated, rehydrated, and stained with the Masson's dye solution set (G1006, Servicebio, China) following the manufacturer's instructions. Sections were then rinsed with 1% glacial acetic acid, dehydrated with two cups of anhydrous ethanol, soaked in 100% ethanol for 5 min, and treated with xylene for 5 min. Finally, the slides were sealed with neutral gum. Collagen deposition morphology was observed under a microscope.

2.6 Terminal deoxynucleotidyl transferase dUTP nick end labeling (TUNEL) staining

Paraffin sections underwent dewaxing, hydration, antigen repair, and cell membrane treatment. Reagent 1 (TdT) and reagent 2 (dUTP) from a TUNEL kit (11684817910, Roche) were mixed in a 2:29 ratio and applied to the tissue. Sections were incubated at 37 °C for 2 h in a wet box. A 3% hydrogen peroxide solution prepared with methanol was used to block endogenous peroxidase, followed by incubation with reagent 3 (converter POD) for 30 min at 37 °C. DAB colorimetric solution (K5007, DAKO) was used, and staining was controlled under a microscope. Positive results were indicated by a brownish-yellow color in the nucleus. Sections were rinsed with

tap water to terminate staining, followed by Harris hematoxylin staining for 3 min, 1% hydrochloric acid alcohol differentiation, a tap-water rinse, bluing with ammonia water, and, finally, dehydration and sealing for microscopic observation.

2.7 DHE staining

Dihydroethidium (DHE) is the most common fluorescent probe detecting intracellular superoxide anion levels and ROS. This dye can freely enter cells and is dehydrogenated under the action of superoxide anions to form ethidium bromide, which can bind to RNA or DNA and produce red fluorescence. When the level of superoxide anions in the cell is high, more ethidium bromide is produced, resulting in stronger red fluorescence and vice versa. Slices were dewaxed and covered with citric acid repair solution. They were placed in a microwave repair box and heated on high for 5 minutes. DHE (PBS: DHE = 200:1–300:1) (D7008, Sigma) was incubated under dark conditions at 37 °C for 30 min. The slides were washed three times with PBS (pH 7.4) on a shaker in the dark for 5 min each time. After slightly drying the slices, DAPI (C1002, Beyotime) staining solution was added at room temperature, avoiding light for 10 min. Slices were sealed with an antifluorescence quenching sealing agent and observed under an inverted fluorescence microscope. Image intensity was analyzed using ImageJ.

2.8 Transmission electron microscopy (TEM)

Liver and colon tissues were cut into 1 × 1 × 2 mm pieces. Tissues were respectively prefixed with 2.5% glutaraldehyde (P1126, Solarbio, Beijing, China) and postfixed with 1% osmium tetroxide (18456, Ted Pella Inc., Redding, CA, USA). After prefixing, postfixing, dehydration, immersion and embedding, samples were cut into 70-nm sections. Double staining with uranyl acetate and lead citrate was employed for TEM observation and capture (HITACHI, HT7800, Tokyo, Japan).

2.9 Transcriptome analysis

Liver samples were flash-frozen in liquid nitrogen and stored at –80 ℃ before sequencing. RNA extraction, quantification, and qualification were followed by cDNA library construction with 370–420 bp fragments purified using the AMPure XP system (Beckman Coulter, Beverly, USA). The library preparations were sequenced on an Illumina NovaSeq platform, and 150-bp paired-end reads were generated. Clean data (clean reads) were obtained by removing reads containing adapters, reads containing poly-N, and low-quality reads from the raw data. At the same time, the Q20, Q30, and GC contents of the clean data were calculated. All downstream analyses were based on clean data with high quality. After read-mapping, featureCounts v1.5.0-p3 was used to count the read numbers mapped to each gene. Then, the FPKM (Fragments per kilobase of exon model per million mapped fragments) of each gene was calculated based on the length of the gene and read-count-mapped to this gene. Differential expression analysis of each pair of groups was performed using the DESeq2 R package (1.20.0). Genes with an adjusted *p*-value ≤ 0.05 were considered differentially expressed. Gene Ontology (GO) and Kyoto Encyclopedia of Genes and Genomes (KEGG) enrichment analyses of differentially expressed genes were implemented using the clusterProfiler R package.

After removing low-quality reads and adapter filtering, the raw data were compared with the mouse reference genome (GRCm39, v.104, https://ftp.ensembl.org/pub/release-104/fasta/mus_musculus/dna) using STAR software (v.2.7.7a, https://github.com/alexdobin/STAR). BAM files were obtained, and the genes of each sample were quantified using FeatureCount software (https://subread.sourceforge.net/featureCounts.html). Sample correlation analysis was performed using Corrplot (v.0.92, https://cran.r-project.org/web/packages/corrplot/vignettes/corrplot-intro.html) to calculate correlation coefficients between samples. The differentially expressed genes between groups were analyzed using DESeq2 (v.1.38.3, https://github.com/thelovelab/DESeq2); an absolute Log2FoldChange value greater than one and an adjusted *p*-value less than 0.05 indicated a significant difference

between groups. A gene heat-map was drawn using pheatmap (v.1.0.12, https://www.rdocumentation.org/packages/pheatmap/versions/1.0.12/topics/pheatmap), and a volcano map of the differentially expressed genes was analyzed and drawn using ggpubr (v.0.6.0,https://rpkgs.datanovia.com/ggpubr). The GO and KEGG analyses of differentially expressed genes were performed using ClusterProfiler, and gene set variation analysis between the groups was performed using GSVA (v.1.46.0, https://bioconductor.org/packages/release/bioc/html/GSVA.html).

2.10 Mitochondrial protein extraction

Mitochondrial proteins were extracted from the liver tissues and AML-12 cells using a mitochondrial isolation kit (EX2620, EX2610, Solarbio) according to manufacturer's indications.

2.11 Cell counting kit-8 (CCK-8) assay

The cell density was adjusted to 3×10^4 cells/m/well in 96-well plates. After certain treatments, the Cell Counting Kit-8 (C0038, Beyotime, China) assay was used for cell viability determination. The working solution was added to the wells for 1 h of incubation and then measured using a microplate reader at 450 nm absorbance.

2.12 Measurement of TG in Cultured cell

TG levels were measured using the Triglyceride Content Detection Kit (BC0625, Solarbio). A 1 mg/mL triglyceride standard solution was prepared by adding 5 mL of reagent 1 to the standard sample. Then, 5–10 million AML-12 cells were collected. After centrifugation, the supernatant was discarded and 1 mL of reagent 1 was added to the cells. Crushing with ultrasound was then applied for 1 min (power 200 W, ultrasound for 2 s, stop for 1 s), centrifugated for 10 min (8000 ×*g*, 4 °C), and the supernatant retained for TG detection. The reader was preheated for 30 min, the wavelength was set to 420 nm (BioTek, Synergy H1), and the TG content was converted to mg/10^4 cell according to the formula in the instruction manual.

2.13 Reactive Oxygen Species (ROS) and MitoSOX^{TM} Detection

The predominant ROS in mitochondria is mitochondrial superoxide, which is readily revealed by the MitoSOX™ Red reagent. The transfected cells were covered in 1 mL (5μM) MitoSOX^{TM} reagent working solution (M36008, Invitrogen, USA) for 10 min at 37 °C in the dark and immediately observed under a confocal microscope (Nikon, AXR).

Intracellular ROS was labeled using the fluorescence probe, 2', 7'-dichlorodihydrofluorescein (DCFH-DA) (S0033S, Beyotime, China). Transfected cells were incubated with 10 μM DCFH-DA probe for 30 min at 37 °C in the dark, then rinsed three times with the medium and evaluated using a confocal microscope.

2.14 Mitochondrial membrane potential assay with JC-1

The mitochondrial membrane potential (ΔΨm) was quantified using JC-1. The decrease in mitochondrial membrane potential, that is, the transition of JC-1 from red (J-aggregates) to green (monomers) fluorescence, is a hallmark event in the early stage of apoptosis. After treatment with 1 mL diluted JC-1 working reagent at 37 °C for 20 min in an incubator (C2006, Beyotime, China), the transfected cells were transferred to fresh medium after washing three times with 1X JC-1 staining buffer. Finally, the distribution of JC-1 was evaluated using confocal microscopy.

2.15 Mitochondrial distribution labeled by Mito-Tracker Red

MitoTracker Red, a green fluorescent probe, can be used for mitochondria-specific fluorescence in living cells. The transfected cells were incubated in a pre-warmed 37 °C staining solution containing 200 nM MitoTracker Green probe (C1035, Beyotime) for 30 min. Following incubation, the samples were washed thrice to remove the residual probe. Next, cells were stained with 1X Hoechst staining solution for live cells (C1027, Beyotime, China) for 10 min, which binds to the cell nucleus, and rinsed with medium droplets three times. The cells were observed to evaluate the mitochondrial distribution using a confocal microscope.

3 Supplementary Results

3.1. Analysis of the PCOS phenotype in a PAMH-induced PCOS mouse model

Two months after birth, we conducted GTT and ITT on control and PAMH mice. No significant differences in blood glucose levels (fasting state and 15 min, 30 min, 60 min, 90 min, and 120 min after intraperitoneal injection of glucose) were observed among the mice at various time points of glucose tolerance testing (p = 0.9124, $p = p$ = 0.9965, p = 0.8113, p = 0.9791, p = 0.6572, and p = 0.9021, respectively) (Figure S1A). However, the area under the curve (AUC) of the GTT was higher in the PAMH group than in the control group (p = 0.0039) (Figure S1C). The decrease in blood glucose levels 30 min after intraperitoneal insulin injection in PAMH mice was much lower than that in control mice ($p < 0.0001$). At 45 and 60 min after insulin injection, blood glucose levels in PAMH mice were higher than those in the normal group (p = 0.0258 and p = 0.0405, respectively) (Figure S1B), and the AUC of the ITT was slightly higher in the PAMH group than in the control group (p = 0.0101) (Figure S1D). These results indicated that insulin resistance and abnormal glucose metabolism were already present in PAMH mice 2 months after birth.

In addition, we compared the GTT (Figure S1E) and ITT (Figure S1F) results of the same six PAMH mice at 2 and 4 months after birth. The AUC values of the GTT and ITT curves of 4-month-old PAMH mice were significantly higher than those of 2-month-old PAMH mice (p = 0.0002 and $p < 0.0001$, respectively) (Figure S1G and H); the AUC value of the ITT curve of 4-month-old PAMH mice was more than twice as high as that of 2-month-old PAMH mice (Figure S1H). These results indicate that 4-month-old PAMH-induced PCOS mice have a more severe phenotype of insulin resistance than 2-month-old mice and that the abnormal phenotype of glucose metabolism in PAMH mice becomes more severe with age.

Figure S1I–T illustrates changes in the GTT and ITT results of the same three PAMH mice before and after Met treatment. After two months of high- and low-dose treatment, no statistically significant difference was observed in the area under the

GTT and ITT curves compared with that before treatment. However, after 2 months of moderate-dose treatment, the AUC values of GTT and ITT in PAMH mice showed a significant decrease (p = 0.0140 and p = 0.0009, respectively).

3.2. Exploration of DHEA and FFA-induced hepatocyte injury

We conducted a series of cellular experiments to explore the optimal rescue concentration and time of Met in cell models of alpha mouse liver-12 (AML-12) hepatic steatosis induced by DHEA and FFA. According to previous literature[2–7], we set up five different concentrations of Met intervention (12.5, 25, 50, 75, and 150 μM) and tested the cells at three time points (12, 24, and 48 h). The addition of different Met concentrations alone to normal cells had almost no negative effect on cell viability. Intervention with palmitic acid (125 μM) and oleic acid (250 μM) for 48 h reduced cell viability and significantly induced cell steatosis, but none of the five Met intervention concentrations significantly improved cell viability (Figure S2E–G). Based on the results, the induction conditions of our FFA model were 125 μM palmitic acid and 250 μM oleic acid for 48 h, with a Met intervention concentration of 10 μM. In addition, we explored the conditions in a DHEA-induced AML-12 hepatocyte steatosis model. When the intervention concentration of DHEA was 50 nM and the intervention time was 48 h, cell steatosis was induced using the lowest DHEA dose (Figure S2H, I).

3.3. Metformin rescues the mitochondrial autophagic level and mitochondrial function of damaged liver cells

We measured and assessed the intracellular ATP content, mtDNA copy number, protein expression of key molecules related to mitochondrial autophagy, and mitochondrial membrane potential. The decrease in cellular ATP content and mtDNA copy number in DHEA and FFA models was partially rescued by Met treatment (Figure 6A, B, E, F). In the DHEA- and FFA-induced cell steatosis models, the expression of Ethe1 decreased by half compared to that in control cells. Similarly, the protein expression of other main molecules related to mitochondrial autophagy (Mfn2,

PINK1, Parkin, and TOMM20) decreased, whereas the expression of the mitochondrial fission protein DRP1 increased (Figure 6C, D, G, H). In addition, immunofluorescence staining was used to observe the co-localization of mitochondria with LC3 and PINK1 using confocal microscopy. Mander's co-localization coefficients indicate the proportion of mitophagy occurring in cells. The results showed that the overlap coefficient of LC3 and PINK1 co-localization with MitoTracker Red in the model group decreased, indicating a reduction in mitochondrial autophagy in cells, whereas Met treatment increased mitochondrial autophagy (Figure 6I, K, L). The mitochondrial membrane potential was evaluated using the mitochondrial probe JC-1. Our findings indicated a substantial increase in green fluorescence and a decrease in red fluorescence in DHEA and FFA groups under confocal microscopy, whereas Met treatment effectively increased the average intensity of red fluorescence (Figure 6J and Figure S2R).

References

[1] Y. Xie, S. Chen, Z. Guo, Y. Tian, X. Hong, P. Feng, Q. Xie, Q. Yu, Down-regulation of Lon protease 1 lysine crotonylation aggravates mitochondrial dysfunction in polycystic ovary syndrome, MedComm (2020) 4 (2023) e396. https://doi.org/10.1002/mco2.396.
[2] A.K. Madiraju, D.M. Erion, Y. Rahimi, X.-M. Zhang, D.T. Braddock, R.A. Albright, B.J. Prigaro, J.L. Wood, S. Bhanot, M.J. MacDonald, M.J. Jurczak, J.-P. Camporez, H.-Y. Lee, G.W. Cline, V.T. Samuel, R.G. Kibbey, G.I. Shulman, Metformin suppresses gluconeogenesis by inhibiting mitochondrial glycerophosphate dehydrogenase, Nature 510 (2014) 542–546. https://doi.org/10.1038/nature13270.
[3] T. Ma, X. Tian, B. Zhang, M. Li, Y. Wang, C. Yang, J. Wu, X. Wei, Q. Qu, Y. Yu, S. Long, J.-W. Feng, C. Li, C. Zhang, C. Xie, Y. Wu, Z. Xu, J. Chen, Y. Yu, X. Huang, Y. He, L. Yao, L. Zhang, M. Zhu, W. Wang, Z.-C. Wang, M. Zhang, Y. Bao, W. Jia, S.-Y. Lin, Z. Ye, H.-L. Piao, X. Deng, C.-S. Zhang, S.-C. Lin, Low-dose metformin targets the lysosomal AMPK pathway through PEN2, Nature 603 (2022) 159–165. https://doi.org/10.1038/s41586-022-04431-8.
[4] H. Fikry, L.A. Saleh, O.A. Mohammed, A.S. Doghish, E.G.E. Elsakka, A.A. Hashish, J. Alfaifi, M.M.S. Alamri, M.I.E. Adam, M.A. Atti, F.A. Mahmoud, H.A.A. Alkhalek, Agmatine alleviates diabetic-induced hyposalivation in rats: A histological and biochemical study, Life Sci 359 (2024) 123220. https://doi.org/10.1016/j.lfs.2024.123220.
[5] C.C. Ikewuchi, J.C. Ikewuchi, M.O. Ifeanacho, Restoration of plasma markers of

liver and kidney functions/integrity in alloxan-induced diabetic rabbits by aqueous extract of Pleurotus tuberregium sclerotia, Biomed Pharmacother 95 (2017) 1809–1814. https://doi.org/10.1016/j.biopha.2017.09.075.
[6] Acute treatment with metformin improves cardiac function following isoproterenol induced myocardial infarction in rats - PubMed, (n.d.). https://pubmed.ncbi.nlm.nih.gov/23406758/ (accessed January 30, 2025).
[7] A.B. Nair, S. Jacob, A simple practice guide for dose conversion between animals and human, J Basic Clin Pharm 7 (2016) 27–31. https://doi.org/10.4103/0976-0105.177703.
